# Variety-dependent accumulation of glucomannan in the starchy endosperm and aleurone cell walls of rice grains and its possible genetic basis

**DOI:** 10.5511/plantbiotechnology.23.0809a

**Published:** 2023-12-25

**Authors:** Ryszard Zamorski, Kei’ichi Baba, Takahiro Noda, Rimpei Sawada, Kana Miyata, Takao Itoh, Hanae Kaku, Naoto Shibuya

**Affiliations:** 1 National Institute of Agrobiological Resources, Ministry of Agriculture, Forestry and Fisheries, Tsukuba, Ibaraki 305-8634, Japan; 2 National Food Research Institute, Ministry of Agriculture, Forestry and Fisheries, Tsukuba, Ibaraki 305-8642, Japan; 3 Faculty of Agriculture and Biotechnology, University of Science and Technology, Bydgoszcz 85-796, Poland; 4 Wood Research Institute, Kyoto University, Uji, Kyoto 611-0011, Japan; 5 Hokkaido Agricultural Research Center, NARO, Memuro, Hokkaido 082-0081, Japan; 6 Plant Biotechnology Laboratory, Life Science Institute, Mitsui Toatsu Chemicals Inc., Mobara, Chiba 297-0017, Japan; 7 Department of Life Sciences, School of Agriculture, Meiji University, Kawasaki, Kanagawa 214-8571, Japan

**Keywords:** all-or-none trait, cell wall, cellulose synthase-like family, glucomannan, rice endosperm

## Abstract

Plant cell wall plays important roles in the regulation of plant growth/development and affects the quality of plant-derived food and industrial materials. On the other hand, genetic variability of cell wall structure within a plant species has not been well understood. Here we show that the endosperm cell walls, including both starchy endosperm and aleurone layer, of rice grains with various genetic backgrounds are clearly classified into two groups depending on the presence/absence of β-1,4-linked glucomannan. All-or-none distribution of the glucomannan accumulation among rice varieties is very different from the varietal differences of arabinoxylan content in wheat and barley, which showed continuous distributions. Immunoelectron microscopic observation suggested that the glucomannan was synthesized in the early stage of endosperm development, but the synthesis was down-regulated during the secondary thickening process associated with the differentiation of aleurone layer. Significant amount of glucomannan in the cell walls of the glucomannan-positive varieties, i.e., 10% or more of the starchy endosperm cell walls, and its close association with the cellulose microfibril suggested possible effects on the physicochemical/biochemical properties of these cell walls. Comparative genomic analysis indicated the presence of striking differences between *OsCslA12* genes of glucomannan-positive and negative rice varieties, Kitaake and Nipponbare, which seems to explain the all-or-none glucomannan cell wall trait in the rice varieties. Identification of the gene responsible for the glucomannan accumulation could lead the way to clarify the effect of the accumulation of glucomannan on the agronomic traits of rice by using genetic approaches.

## Introduction

Plant cell wall is a complicated cell surface structure consisting of various polysaccharides, glycoproteins and, in the case of secondary walls, lignin. Plant cell wall plays important roles in the regulation of plant growth, development and responses to environmental changes (Nishitani and Demura 2015; Zhang et al. 2021). For human beings, plant cell walls are the major source of dietary fiber, and their properties also affect food processing and the quality of end products (Capuano and Pellegrini 2019; Grundy et al. 2016). Plant cell walls, especially secondary walls, are also an important resource for paper/textile industry and sustainable energy supply (Van de Wouwer et al. 2018).

Detailed structural studies of cell wall components and their possible interactions resulted in several models for growing primary cell wall (Carpita and Gibeaut 1993; Cosgrove 2014) and secondary cell wall (Zhong et al. 2019). Changes of the cell wall components/structure during the evolution of land plants have also been discussed (Bulone et al. 2019; Burton and Fincher 2014; Sarkar et al. 2009). On the other hand, knowledge on the genetic variability of cell wall components in a single plant species is still limited. Genetic effect on the structure/properties of secondary cell walls has been studied in relation to the strength of culm, culm lodging and their digestibility (Houston et al. 2015; Kaur et al. 2017; Manga-Robles et al. 2021; Nguyen et al. 2020; Wang et al. 2020). Genetic variation of plant cell walls has also been studied for fruits (Galvez-Lopez et al. 2011; Lahaye et al. 2012; Popovsky-Sarid et al. 2017) and seeds (Bassett et al. 2021; Garcia-Gimenez et al. 2019; Wood et al. 2018), exploring the relationships between agronomic traits and cell wall structure. In a model plant, *Arabidopsis thaliana*, genetic variability of polysaccharide composition as well as possible changes in their structure has been studied for primary cell wall (Mouille et al. 2006) and also for seed mucilage (Voiniciuc et al. 2016), which is a model for the biosynthesis and secretion of cell wall polysaccharides (Arsovski et al. 2010). These studies indicated genetic variability of cell wall components/structure within each plant species, but such variation is mostly limited to the proportion of constituent polysaccharides, or the changes in the structure of some polysaccharides. So far as we noticed, all-or-none distribution of a specific cell wall polysaccharide within a plant species has not been reported.

To obtain insight into the genetic variability of cell wall structure within a major crop, rice, we analyzed the variation of cell wall polysaccharides of starchy endosperm from various rice varieties and found that the starchy endosperm cell walls of rice are clearly classified into 2 groups, depending on the presence/absence of β-1,4-linked glucomannan, which seems to be firmly associated with cellulose microfibril. Immunoelectron microscopic observation showed the presence of glucomannan in the cell walls of starchy endosperm and the middle portion of the aleurone cell walls, indicating the glucomannan was synthesized during the early stage of endosperm development. Significant accumulation of the glucomannan, often more than 10% of the starchy endosperm cell wall, and its close association with cellulose microfibril suggested that the presence of glucomannan could affect the physicochemical properties of the cell wall and eventually some biological aspects of rice grains as well as their characteristics as a food/industrial material.

## Materials and methods

### General analytical methods

Total carbohydrate was determined by phenol-sulfuric acid method. Protein content was analyzed by the Bradford or Lowry method.

### Chemicals

Cellotetraose and cellopentaose were purchased from Seikagaku Co. (Tokyo). Glc-(β-1,4)-Man was kindly provided by Dr. Isao Kusakabe of the University of Tsukuba.

### Rice samples

Brown rice grains of the varieties used in this work were obtained through the breeding laboratories of the regional research centers of Ministry of Agriculture, Forestry and Fisheries of Japan and the prefectural research centers. Part of the rice seeds were also obtained from the Gene Bank of National Institute of Agrobiological Sciences.

### Preparation of starchy endosperm cell walls

Starchy endosperm cell walls were prepared based on the modification of previously reported method (Shibuya et al. 1985). Starch granules were removed by aqueous suspension filtration method, instead of previously described dimethyl sulfoxide extraction, to minimize the loss of relatively soluble cell wall polysaccharides. Macro/semi-microscale preparation method, described as follows, was applied depending on the purpose of the experiment.

#### (1) Macro-scale preparation of the cell wall for fractionation and characterization of cell wall polysaccharides

Brown rice grains were milled with a Satake motor one-pass test mill (Type SKD) to 80% yield. Milled rice grains were ground with a Brabender laboratory mill (OHG Duisburg) to obtain milled rice flour. Fifty grams of the milled rice flour were treated with boiling 80% methanol (6 ml/g flour) for 1 h to inactivate enzymes and to remove lipids. After cooling, the suspension was filtered through a glass-beaded funnel, washed with cold methanol, then with acetone and finally air-dried. The defatted flour was treated with 1% SDS-mercaptoethanol solution (10 ml/g flour) overnight with intense stirring. Released/solubilized starch and proteins were washed out with distilled water through a metal testing sieve No.45 (Sanpo Co., Ltd. Japan) until no starch was detected in the filtrate by iodine-potassium iodide staining. The crude cell wall preparation recovered from the sieve was transferred to a 100 ml erlenmeyer flask, adjusted to 50–60 ml with distilled water and homogenized with a Physcotron homogenizer (Microtech Co., Ltd., Japan) for 20–40 min in a cold room. Starch granules liberated from the cell walls were filtered off as described before. This treatment was repeated until no starch granule was detected microscopically in the cell wall preparation by iodine-potassium iodide staining. The cell wall preparation was lyophilized and stored.

#### (2) Semi-microscale preparation of the cell wall for the study of varietal difference

Three grams of brown rice grains were milled to 80% yield with a small scale milling machine (Kett PEARLEST grain polisher, Kett Electric Laboratory, Tokyo). Milled rice grains were then crushed and powdered with mortar and pestle. The milled rice grains were used as a starting material to prepare starchy endosperm cell walls following the similar procedure as described above.

### Preparation of the crude cell wall material from the outer layers of brown rice

Rice germs were carefully removed from brown rice grains with a tweezer and surgical blade. The remaining parts of the grains were rinsed with a mixture of chloroform and methanol (1 : 1). Then the outer layers of the grains were carefully removed by gently agitating the grains with a glass rod in a glass tube containing the same solution. Separated outer layers were collected with a Pasteur pipette, spun down and air-dried. The dry material was directly used as a crude cell wall material for sugar composition analysis.

### Preparation of callus cell walls

Calli derived from rice root cells of japonica varieties, Kitaake and Yukihikari, were kindly supplied by Dr. Kunihiro Kasamo of National Food Research Institute. Other calli used in this work were induced from mature rice seeds by incubating on solid N6 media supplemented with 2,4-D (2 mg l^−1^) as described by Ozawa (Ozawa 2009). Established calli were transferred to liquid N-6 medium and subcultured weekly as described previously (Tsukada et al. 2002).

Callus cells were homogenized in ice-cold 0.1 M K-phosphate buffer, pH 6.3, for 5 min with a Polytron homogenizer (Kinematica AG, Malters, Switzerland). After centrifugation (2,000×g, 15 min), the pellet was washed twice with the same buffer. The washing process was repeated twice. The resulting solid material was checked for the contamination of cytoplasmic components with acetocarmine solution (Wako Pure Chemicals Co.). Cell wall preparations thus obtained were further washed with distilled water several times and finally lyophilized.

### Fractionation of cell wall polysaccharides

Thirty mg of the starchy endosperm cell wall preparation was extracted three times with 4 ml of boiling water for 2 h. Solubilized polysaccharides were recovered by spinning down the insoluble materials and combined. The soluble fraction was dialyzed against distilled water and lyophilized to give “hot water-soluble fraction”. Remaining insoluble materials were further extracted with hot 0.25% ammonium oxalate solution as similar to the boiling water extraction and recovered as “hot oxalate soluble fraction” after dialysis and lyophilization. Remaining residues were further extracted with 4 M NaOH/0.1% NaBH_4_ solution at room temperature. The NaOH extract was neutralized with acetic acid, dialyzed against distilled water and recovered by lyophilization as “4 M NaOH-soluble fraction”. Finally, the residues recovered after NaOH extraction was thoroughly washed with distilled water and recovered by lyophilization. This fraction was named as “α-cellulose fraction”.

### Purification of glucomannan from α-cellulose fraction

α-Cellulose fraction prepared from var. Eiko or Kitaake was extracted with 0.81 M sodium tetraborate/0.013 M sodium borohydride in 6 M NaOH for 2 h (Lindberg 1977). This extraction was repeated three times. Supernatants containing crude glucomannan were recovered by centrifugation, neutralized with acetic acid and dialyzed against distilled water. Since the crude glucomannan preparation was still contaminated with significant amount of arabinoxylan, it was further digested with a purified endoxylanase preparation from *Streptomyces olivaceoviridis E-86* (kindly supplied by Dr. Isao Kusakabe of the University of Tsukuba) in 10 mM phosphate buffer, pH 6.3, overnight at room temperature. The reaction mixture contained 4 U of the xylanase, where 1 U of xylanase was defined as the amount of the enzyme that liberates 870 µg xylose from xylan substrate in 30 min. After the xylanase treatment, purified glucomannan was recovered by centrifugation, washed several times with water and lyophilized.

### Acid hydrolysis of cell wall preparation/polysaccharide components and HPAEC analysis of neutral sugars

Cell wall preparations or purified polysaccharides were hydrolyzed by two-step sulfuric acid hydrolysis (Saeman et al. 1945) as follows. These samples were pretreated with 72% sulfuric acid for 1 h, then, hydrolyzed with diluted 0.5 M sulfuric acid at 121°C for 1 h in screw-capped vials. The hydrolysate was neutralized by passing through a small column of Amberlite CG 400 (acetate form), eluted with distilled water and concentrated by evaporation. Neutral sugars were analyzed by high-performance anion-exchange chromatography (HPAEC), using a Dionex BioLC equipped with a CarboPac PA1 column and a pulsed amperometric detector. The column was equilibrated with 0.1 M NaOH for 30 min before each analysis. After the application of the sample, the column was eluted with 0.005 M NaOH for 5 min and then with water for 30 min.

### Partial acid hydrolysis of (gluco)mannan

Purified rice glucomannan or ivory nut mannan, which was kindly supplied by Dr. Akira Misaki of Osaka City University, was partially hydrolyzed by heating with 90% formic acid at 70°C for 30 min, followed by heating with 0.5 M trifluoroacetic acid (TFA) at 90°C for 1 h. Hydrolyzed samples were concentrated to dryness after each step and then redissolved with water. The partial acid hydrolyzates were subjected to gel filtration or HPAEC.

For gel filtration analysis samples were added to a connected column of Bio Gel P-2 (2.6×88 cm) and P-4 (2.6×77 cm) and eluted with distilled water. Fractions were collected and analyzed for carbohydrate by phenol-sulfuric acid method. Disaccharide fraction obtained from the gel filtration and standard sugars were analyzed by HPAEC as follows. After the application of the sample, the column was eluted with 0.1 M NaOH for 5 min, followed by a linear gradient of 0 to 0.1 M sodium acetate in 0.1 M NaOH for 30 min.

### Methylation analysis

Purified glucomannan was methylated by Hakomori method (Hakomori 1964). Briefly, purified glucomannan was dissolved with dimethyl sulfoxide and methylated with methylsulfinyl carbanion and methyl iodide. After the reaction was completed, the mixture was dialyzed against distilled water, concentrated under reduced pressure and the methylated glucomannan was extracted with chloroform-methanol (1 : 1). Methylated glucomannan was then hydrolyzed by heating with 90% formic acid at 100°C for 1 h and then 1 M TFA at 121°C for 1 h. Partially methylated sugars thus obtained were reduced with NaBH_4_ and converted to the corresponding alditol acetates by heating with acetic anhydride and pyridine (1 : 1). Partially methylated alditol acetates were analyzed by GC and GC-MS as described previously (Yamaguchi et al. 2000).

### Preparation of mannotetraosyl-BSA for immunization

Partial acid hydrolysate of ivory nut mannan, which was prepared as described in the previous section, was fractionated by gel filtration chromatography on Bio Gel P-2 and P-4 as described. Fractions containing mannopentaose were collected and lyophilized. Purified mannopentaose was coupled with bovine serum albumin (BSA) by reductive amination. Briefly, mannopentaose (2 mg) was incubated in 0.2 M phosphate buffer (pH 8.8, 0.5 ml) containing BSA (7 mg) and NaCNBH_3_ at 25°C for 24 h. The mannotetraosyl-BSA (Man_4_-BSA) was purified by gel filtration chromatography on a Bio Gel P-2 column (0.9×50 cm) by eluting with 10 mM PBS (pH 7.2). Determination of total carbohydrate and protein in the Man_4_-BSA preparation indicated that the final preparation contained 5.6 mol of intact mannotetraosyl residues per one mole of BSA.

### Preparation of cellotetraosyl- and mannooligosyl-Sepharose for affinity purification of antibody

Mannooligosaccharides mixture (DP=2 to 9) or cellopentaose was dissolved in 10 mM phosphate buffer (pH 9.6) and coupled to epoxy-activated Sepharose 6B (Pharmacia Biotech). After incubation at 25°C for 17 h, the Sepharose beads were washed with the same buffer and uncoupled residues were blocked by 1 M glycine solution at room temperature for 2 h. The amount of coupled sugars in the final products, mannooligosyl-Sepharose 6B (Man_(oligo)_-Sepharose) and cellotetraosyl-Sepharose 6B (Cello_4_-Sepharose), were 0.75 mg mannooligosaccharides/ml gel and 1.9 mg cellotetraose/ml gel, respectively.

### Preparation of mannooligosaccharide-specific antibody

Α β-1,4-linked mannooligosaccharide-specific antibody was prepared based on the method previously reported for anti-α-L-arabinofuranosyl antibody (Misaki et al. 1988). Rabbit antiserum against Man_4_-BSA was obtained by subcutaneous injection of Man_4_-BSA in Freund’s complete adjuvant (DIFCO Lab., Detroit). Mannooligosaccharide-specific antibody (Man_4_-antibody) was purified from the antiserum by successive affinity chromatography on Cello_4_- and Man_(oligo)_-Sepharose columns. Briefly, the antiserum was applied to a Cello_4_-Sepharose column (0.9×6 cm) to remove antibody against cellooligosaccharides, if present. The unbound fraction was then applied to a column of Man_(oligo)_-Sepharose (0.9×6 cm). After the column was washed with 50 mM borate buffered saline (pH 8.5), Man_4_-antibody was eluted with 0.17 M glycine-HCl buffer into a tube containing 1/10 elute volume of 1 M tris solution.

### Characterization of the mannooligosaccharide-specific antibody by ELISA

Enzyme-linked immunosorbent assay (ELISA) for the characterization of the Man_4_-antibody was carried out as follows. A 96-well flat bottom microtiter plate was coated with Man_4_-BSA (2 µg/100 µl/well) in 0.1 M carbonate buffer (pH 9.6) overnight at 4°C. After blocking with 1% ovalbumin (OVA) in the same buffer, the wells were washed 3 times with PBS-0.1% tween 20. A 50 µl aliquot of the serially diluted Man_4_-antibody (PBS/0.1% OVA/0.1% tween 20) was added to each well and incubated at 37°C for 2 h. After the wells were washed thoroughly with PBS-0.1% tween 20, the antibody reacted with the Man_4_-BSA was detected with the use of horse radish peroxidase-labeled goat anti-rabbit IgG (1/2,500 dilution, Sigma Chemical Co.). After the addition of substrate (2,2′-azino-bis(3-ethylbenzothiazoine-6-sulfonic acid) diammonium salt), color intensity at 415 nm was determined by a microplate reader. Hapten inhibition experiments were performed by the addition of the increasing amount of mono- and oligosaccharides to the microtiter wells.

### Immunoelectron microscopy

Maturing rice seeds of Fujisaka 5 (glucomannan-containing variety) and Reishikou (glucomannan-less variety) were harvested 7 days after flowering and used for immunoelectron microscopic observation as described previously (Baba et al. 1994). Briefly, dehulled rice seeds were cut into three pieces and immediately fixed with 1.5% paraformaldehyde and 2.5% glutaraldehyde in PIPES buffer (pH 7.4) at 25°C for 8 h. The samples were washed 3 times with 20 mM sodium-cacodylate buffer, pH 7.4 and postfixed with 1% OsO4 in cacodylate buffer at 4°C overnight. The fixed samples were dehydrated with increasing concentration of ethanol (25, 50, 75, 90%/cacodylate buffer, and finally 100% ethanol). Dehydrated samples were treated stepwisely with increasing concentration of LR White resin (London Resin Co., Ltd., Hampshire, England; resin/ethanol, 1 : 2, 2 : 1 and 100% resin). Finally, the samples were embedded in the 100% resin at 50°C overnight. Ultrathin sections were obtained by cutting with an ultramicrotome and mounted on 150 mesh copper grids supported by polyvinyl formal (Nacalai Tesque) film. The ultrathin sections on the grids were pretreated with 1% OVA in 10 mM PBS (pH 7.2) for 30 min and then incubated with the Man_4_-antibody in 0.1% OVA/0.05% Tween 20/PBS at room temperature for 1 h. Each grid was then washed 10 times with PBS containing 0.05% Tween 20 (this rinsing was repeated after all the following steps) and reacted with protein A-colloidal gold at room temperature for 30 min. After rinsed successively with the same buffer and distilled water, the ultrathin sections were air-dried and stained with uranyl acetate for 20 min. Deposition of colloidal gold was observed with an electron microscope.

Hapten inhibition experiments for the immunoelectron microscopy were performed by the addition of mannooligosaccharides mixture (DP=2 to 9) to the Man_4_-antibody. Negative controls contained pre-immune serum and PBS instead of Man_4_-antibody.

### Comparative genomic analysis of cellulose/mannan synthase-related genes on rice chromosome 9 between Kitaake and Nipponbare

Genes related to cellulose synthase and mannan synthase were searched by using The Rice Annotation Project Database (RAP-DB, https://rapdb.dna.affrc.go.jp/ (Accessed May 22, 2023)) (Sakai et al. 2013). Four cellulose synthase(-like) genes were found on rice chromosome 9 by using the query “cellulose synthase”; Os09g0422500, Os09g0478300, Os09g0428000, Os09g0478100. The amino acid sequences of these gene products were used for the BLAST search of Kitaake genome by using *Oryza* sativa Kitaake v3.1 database in Phytozome v13 (https://phytozome-next.jgi.doe.gov/ (Accessed May 22, 2023)). No difference was observed for the whole amino acid sequences of these gene products between Kitaake and Nipponbare.

On the other hand, survey of RAP-DB with the query “mannan synthase” identified a gene located on the chromosome 9, Os09g0572500 (LOC_Os09g39920 in MSU), which was previously reported as an ortholog of *TaCslA12* of wheat and named as *OsCslA12* (Verhertbruggen et al. 2021). Comparison of the cDNA and amino acid sequences of *OsCslA12*/OsCslA12 between Nipponbare and Kitaake indicated the presence of noticeable difference between their sequences (Nipponbare, Os09t0572500-01; Kitaake, OsKitaake09g208700.1). Transmembrane domains of OsCslA12 of Nipponbare and Kitaake, and TaCslA12 were predicted by using Phobius (https://phobius.sbc.su.se/index.html (Accessed May 22, 2023)) (Kall et al. 2007). The expression profiling data of *OsCslA12* in rice tissues/organs were obtained from The Rice Expression Profile Database Version 3.0 (RiceXPro, https://ricexpro.dna.affrc.go.jp/ (Accessed May 22, 2023)).

## Results

### Rice varieties are classified into two groups based on the presence/absence of a mannose-containing polysaccharide in the starchy endosperm cell wall

**Figure 1:**
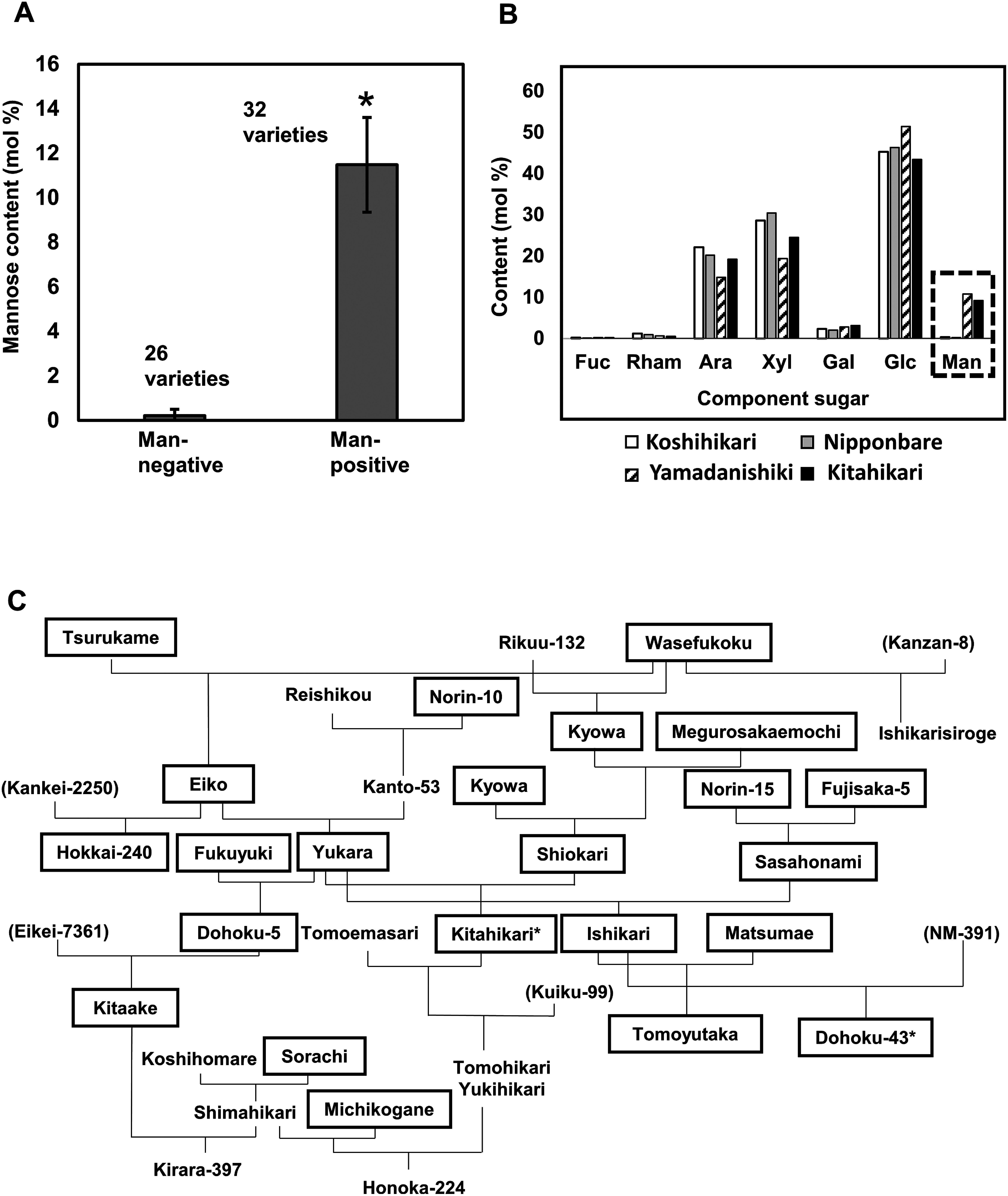
Figure 1. Starchy endosperm cell walls from rice varieties are classified into two groups depending on the presence/absence of mannose as a component sugar. A. Average mannose contents of starchy endosperm cell walls from mannose-less and mannose-containing varieties. Endosperm cell wall preparations from arbitrary collected 58 rice varieties (not including those varieties described in Figure 1C, except Kitahikari and Dohoku 43) were analyzed for their neutral sugar composition after acid hydrolysis. Average mannose contents were 0.22±0.28 and 11.5±2.1% for mannose-less and mannose-containing varieties, respectively. Error bars indicate standard deviation. The asterisk indicates significant difference from the mannose-less varieties by Student’s *t*-test (*p*<0.01). For the detailed distribution of mannose-containing and mannose-less varieties, see Supplementary Figure S1. B. Typical example of neutral sugar composition of starchy endosperm cell walls from mannose-containing and mannose-less rice varieties. Dotted square shows the difference in the mannose content between the two groups. C. Inheritance of mannose-containing cell wall trait among genetically related rice varieties. Solid squares indicate the mannose-containing varieties. Those varieties shown in parentheses were not used for cell wall analysis. Mannose contents were 8.0–12.4% and 0–0.4% for mannose-containing and mannose-less varieties, respectively. Symbol “*” indicates the varieties, “Kitahikari” and “Dohoku-43”, described in the text.

It was previously reported that the starchy endosperm cell wall of rice is a thin, not lignified cell wall and consists of (glucurono)arabinoxylan, β-(1→3, 1→4)-glucan, xyloglucan, rhamnogalacturonan-I type pectic polysaccharide, cellulose and hydoxyproline-containing glycoprotein(s) (Shibuya and Iwasaki 1978, 1985; Shibuya and Misaki 1978; Shibuya and Nakane 1984). Part of the (glucurono)arabinoxylan was suggested to be cross-linked through diferulic acid in the cell wall (Shibuya 1984). To evaluate the possible genetic variation of the cell wall structure, starchy endosperm cell walls were prepared from the rice grains of 58 japonica as well as indica varieties, which were collected arbitrarily based on the availability for the groups shown in Supplementary Figure S1, and their neutral sugar compositions after acid hydrolysis (hereafter referred to as sugar composition) were compared. The results of such analyses, as shown in Figure 1A, B, showed that the starchy endosperm cell walls of rice grains are clearly classified into two groups depending on the presence or absence of mannose as a component sugar. Starchy endosperm cell walls from mannose-less group of rice varieties practically did not contain mannose but those from mannose-containing varieties contained, on average, 11–12% mannose as a neutral sugar (Figure 1A, B and Supplementary Figure S1). These results indicated the presence of variety-dependent accumulation of some mannose-containing polysaccharide(s) in the starchy endosperm cell walls of rice.

Concerning to the distribution of the mannose-containing cell wall among the japonica varieties, it is noteworthy that the japonica varieties specialized for Japanese “Sake” brewing and many upland varieties often contained mannose in the starchy endosperm cell walls (Supplementary Figure S1B, S1D and S1E; It should be noted that the data in Supplementary Figure S1E were previously shown as a part of a review article in Japanese (Shibuya 1993, https://www.jstage.jst.go.jp/article/jbrewsocjapan1988/88/12/88_12_910/_article/-char/ja/). These data were converted to mol% from the peak area ratio in the previous publication and appear slightly different). On the other hand, many modern japonica, non-waxy, paddy varieties bred for preferable eating quality for Japanese people, which give soft and sticky cooked rice, tend not to contain mannose in the starchy endosperm cell walls (Supplementary Figure S1A). To evaluate the inheritance of mannose-containing cell wall trait during the breeding process, we focused on two mannose-containing varieties, Dohoku-43 and Kitahikari, as we could follow the breeding process and obtain the samples for most related varieties. Starchy endosperm cell walls from these varieties were then analyzed for the mannose content. The results of such analyses confirmed the all-or-none distribution of the mannose-containing cell wall in the descendant varieties (Figure 1C). These results and the similar discontinuous distribution of mannose-containing cell wall within other rice varieties (Figure 1A) suggested that it is a qualitative trait controlled by a single (or two) gene(s) (Kumar et al. 2017; St Clair 2010).

### The mannose-containing polysaccharide is a β-1,4-linked glucomannan associated with cellulose microfibril

**Figure 2:**
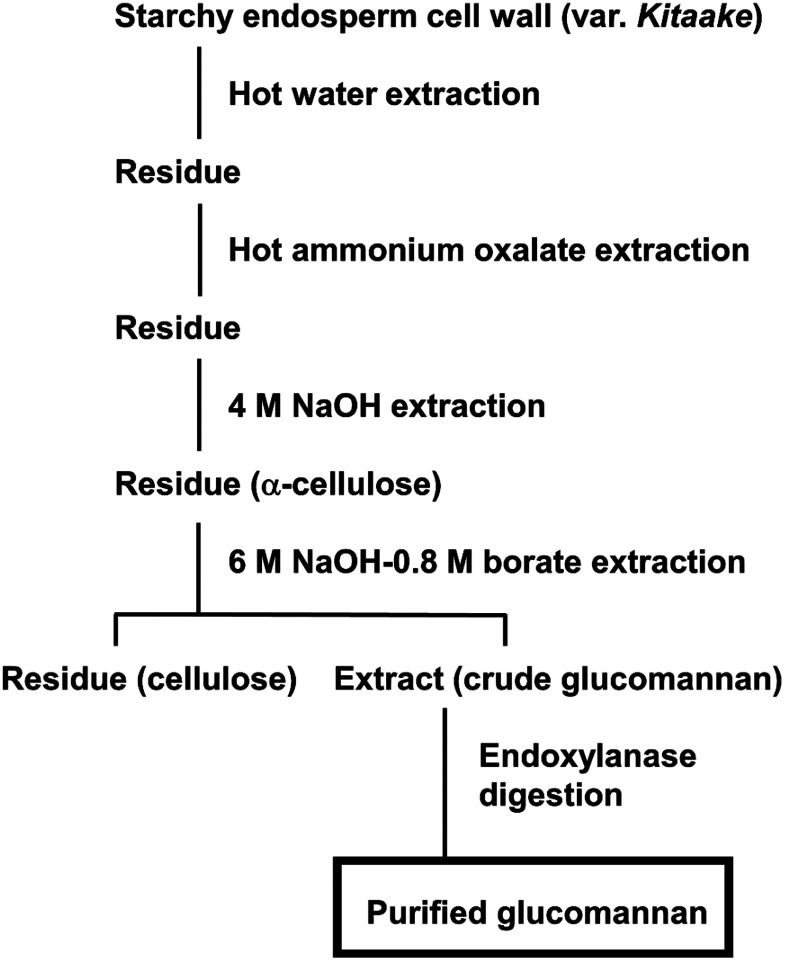
Figure 2. Purification of rice glucomannan. Glucomannan was purified from the starchy endosperm cell wall of var. Kitaake, a mannose-containing rice variety by stepwise extraction and endoxylanase treatment. For details, see “Materials and methods”.

To characterize the mannose-containing polysaccharide in the starchy endosperm cell wall, cell wall polysaccharides in the cell wall from var. Kitaake, a mannose-containing variety, were fractionated by differential extraction method (Figure 2) (Shibuya and Iwasaki 1978; Shibuya et al. 1985) and the sugar composition of each fraction was analyzed by HPAEC after acid hydrolysis. As shown in Table 1, the mannose-containing polysaccharide was not detected in the hot water-soluble and hot oxalate-soluble fractions. Instead, the mannose-containing polysaccharide was enriched in the 4 M NaOH-soluble hemicellulose fraction and in the α-cellulose fraction, which remained after the extraction with 4 M NaOH. Sugar compositions of these fractions indicated that the 4 M NaOH-soluble fraction also contained arabinoxylan, xyloglucan and β-(1→3, 1→4)-glucan as previously reported (Shibuya and Misaki 1978). On the other hand, the α-cellulose fraction was mostly not contaminated with these polysaccharides. Thus, we decided to purify the mannose-containing polysaccharide from the α-cellulose fraction.

**Table 1:** Table 1. Yield and sugar composition of each polysaccharide fraction obtained by stepwise extraction of rice endosperm cell wall.

Fraction	Yield (%)	Sugar composition (mol%)						
		Fuc	Rham	Ara	Gal	Glc	Xyl	**Man**
Hot water extract	11.3	0.4	3.7	21.8	8.3	51.3	14.4	**0**
Hot ammonium oxalate extract	9.3	0.4	2.6	15.0	5.2	62.8	14.0	**0**
4 M NaOH extract	43.1	0.1	0.4	16.0	2.1	38.5	23.9	**19.0**
α-Cellulose	24.3	0	0	2.0	0.3	85.8	3.3	**8.7**
Glucomannan (crude)*	3.7	0	0.4	7.6	1.7	8.6	11.0	**70.8**
Glucomannan (purified)*	1.1	0	0	0.7	1.2	8.0	1.1	**89.1**

A typical result from repeated fractionation experiments is shown here. *Glucomannan preparations were purified from the α-cellulose fraction and their yields were shown as the percentage of the original endosperm cell wall preparation.

The mannose-containing polysaccharide was successfully solubilized from the α-cellulose fraction by the extraction with 6 M NaOH containing 0.8 M borate (Table 1, “Glucomannan (crude)”). As the sugar composition of this preparation still indicated the presence of a small amount of arabinoxylan, this fraction was further digested with the purified endoxylanase from Streptomyces *olivaceoviridis* E-86 (Kuno et al. 1999; Kusakabe et al. 1977; Shibuya 1984; Yoshida et al. 1990). The final preparation was mostly composed of glucose and mannose (Table 1, “Glucomannan (purified)”).

Methylation analysis of the purified polysaccharide showed that both glucose and mannose residues are linked through 1,4-linkage (Table 2), indicating the polysaccharide is a 1,4-linked glucomannan. Detection of a small amount of terminal galactose residue and 4,6-linked mannose and glucose residues indicated that the glucomannan is actually a branched galactoglucomannan having terminal galactose residues attached to mannose or glucose residues through 1,6-linkage, though the frequency of the branching is very low. Disproportion between the amount of terminal and branching residues observed in the methylation analysis (Table 2) could be explained by the under methylation of the insoluble glucomannan preparation as well as the partial loss of the alditol acetate derived from terminal galactose residue because of its higher volatility.

**Table 2:** Table 2. Methylation analysis of the purified glucomannan.

Sugar residue	Linkage	(mol%)
Gal	terminal	1.4
Glc	4-linked	8.6
	4,6-linked	0.8
Man	4-linked	87.1
	4,6-linked	2.1

**Figure 3:**
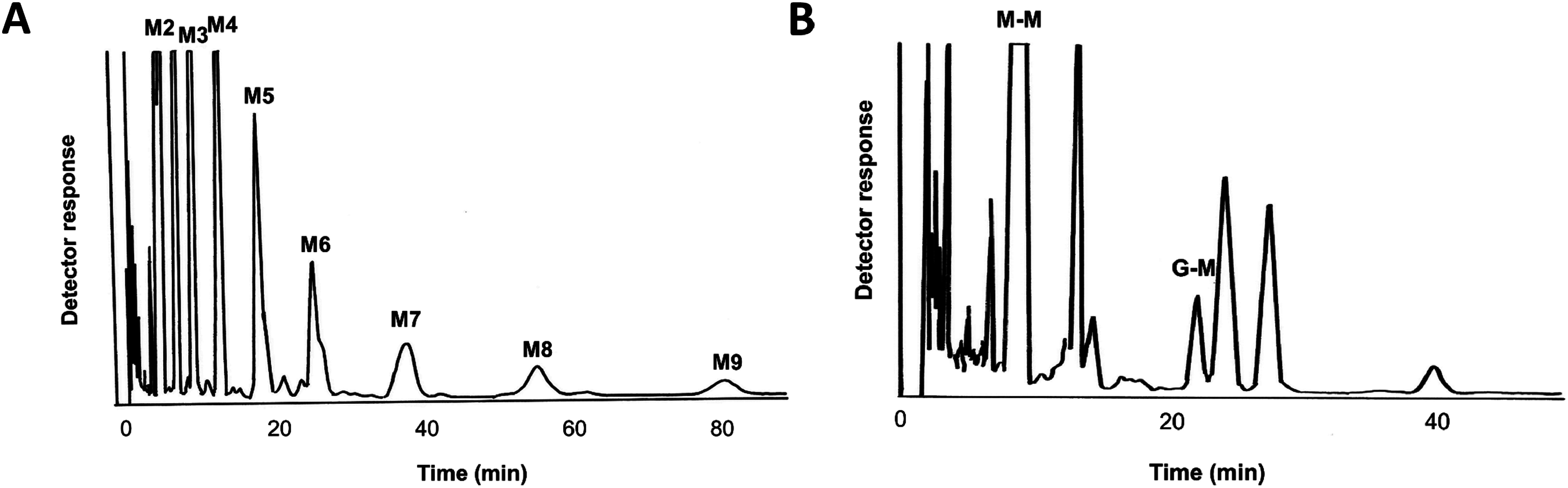
Figure 3. Analysis of the partial acid hydrolysate of rice glucomannan. A. HPAEC profile of the partial acid hydrolysate of the purified glucomannan. Mixture of the partial acid hydrolysate was analyzed by HPAEC. Symbols M_2_–M_9_ indicate the peaks corresponding to the standard β-1,4-linked mannooligosaccharides. B. HPAEC profile of the disaccharide fraction from the partial acid hydrolysate of the glucomannan. Partial acid hydrolysate of the rice glucomannan was first fractionated on a Bio Gel P-2/P-4 column to obtain disaccharide fraction (Supplementary Figure S2). The disaccharide fraction was further analyzed by HPAEC. Symbols M-M and G-M indicate the peaks corresponding to the standard disaccharides, Man-(β1,4)Man and Glc-(β-1,4)-Man, respectively.

To further clarify the structure of the glucomannan, it was partially hydrolyzed by successive heating with 90% formic acid and then 0.5 M TFA. Ivory nut mannan was partially hydrolyzed similarly as a standard polysaccharide. Partial hydrolysates thus obtained were analyzed by HPAEC and compared for their elution profiles. HPAEC profile of the partial acid hydrolysate of rice glucomannan showed the presence of a series of β-1,4-linked mannooligosaccharides (Figure 3A). Furthermore, detailed analysis of the disaccharide fraction, which was obtained by gel filtration of the partial hydrolysate (Supplementary Figure S2), by HPAEC showed the presence of a disaccharide, Glc-(β-1,4)-Man (Figure 3B), confirming the glucose and mannose residues were originated from a polysaccharide, β-1,4-linked glucomannan.

### Immunoelectron microscopic analysis indicated the glucomannan is accumulated in the starchy endosperm and aleurone cell walls during the early development of rice endosperm

**Figure 4:**
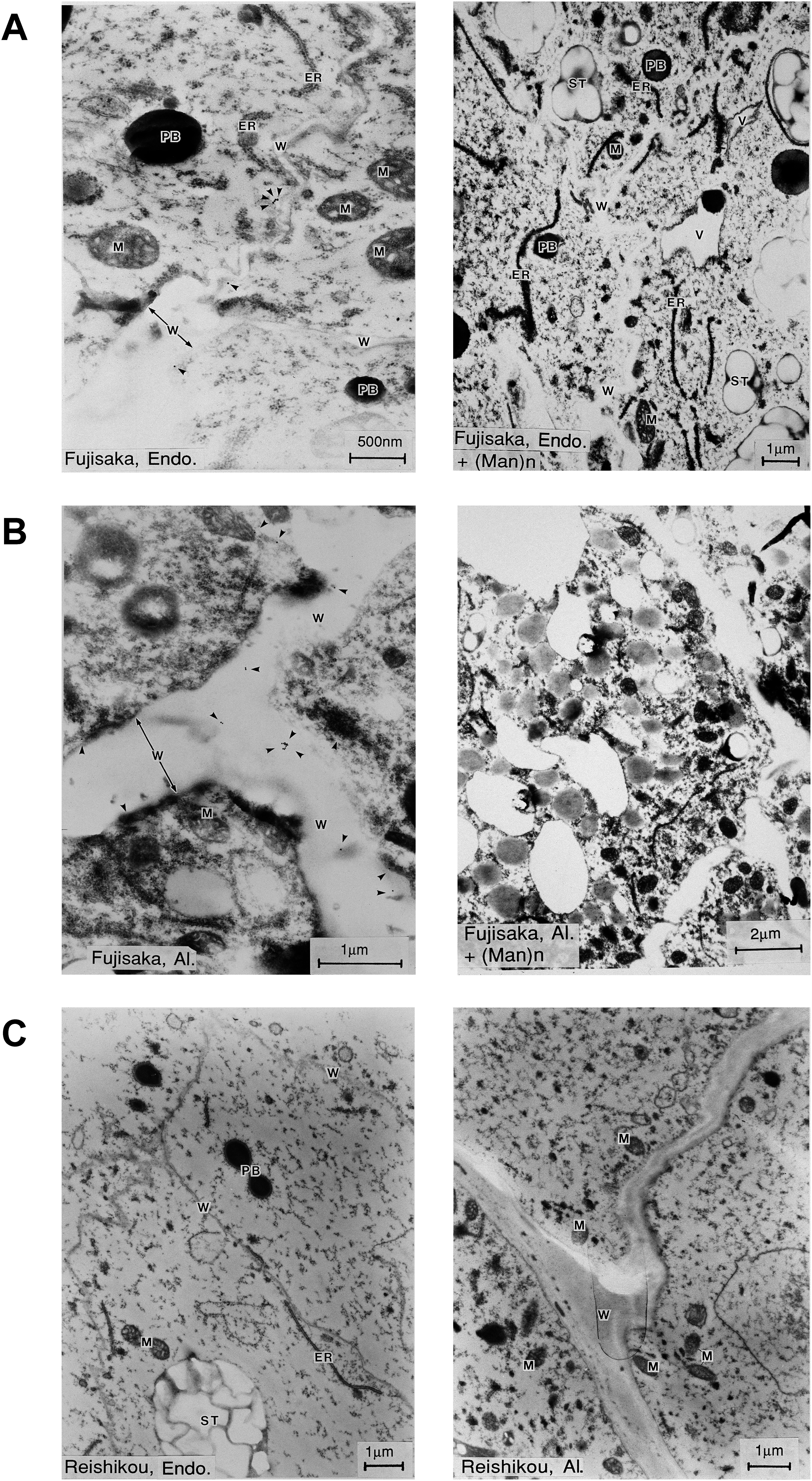
Figure 4. Immunoelectron microscopic analysis of glucomannan in the starchy endosperm and aleurone cell walls of maturing rice grains. A. Starchy endosperm of a glucomannan-containing variety, Fujisaka 5. Left, without mannooligosaccharides; Right, with mannooligosaccharides. B. Aleurone layer of Fujisaka 5. Left, without mannooligosaccharides; Right, with mannooligosaccharides. C. Starchy endosperm (left) and aleurone layer of a glucomannan-less variety, Reishikou. Both tissues were treated with the Man_4_-antibody in the absence of mannooligosaccharides. PB, protein body; ER, endoplasmic reticulum; M, mitochondria; W, cell wall; ST, starch granule; V, vacuole. Arrowheads indicate immune labeling with gold particles.

Accumulation of glucomannan in the maturing rice grain was analyzed by immunoelectron microscopy by using a β-1,4-linked mannooligosaccharide-specific antibody, which was obtained by immunizing a rabbit with mannotetraosyl-BSA glycoconjugate. The purified antibody (Man_4_-antibody) obtained after affinity purification showed a several hundred times higher affinity for β-1,4-linked mannotetraose compared to cellotetraose and cellopentaose (Supplementary Figure S3). Ultrathin sections of developing rice grains from Fujisaka 5 (glucomannan-containing) and Reishikou (glucomannan-less), both harvested at 7 days after flowering, were treated with the Man_4_-antibody and protein A-colloidal gold successively and observed with an electron microscope as previously described for woody materials (Baba et al. 1994). Gold particles were found in the cell walls of both starchy endosperm (Figure 4A, left) and aleurone cells (Figure 4B, left) of Fujisaka 5 but not detected in the corresponding tissues of the glucomannan-less variety, Reishikou (Figure 4C). Immunogold label observed in the cell walls of Fujisaka 5 was completely inhibited by the addition of mannooligosaccharides (Figure 4A, B, right), supporting the specific detection of glucomannan by the immunoelectron microscopy.

**Figure 5:**
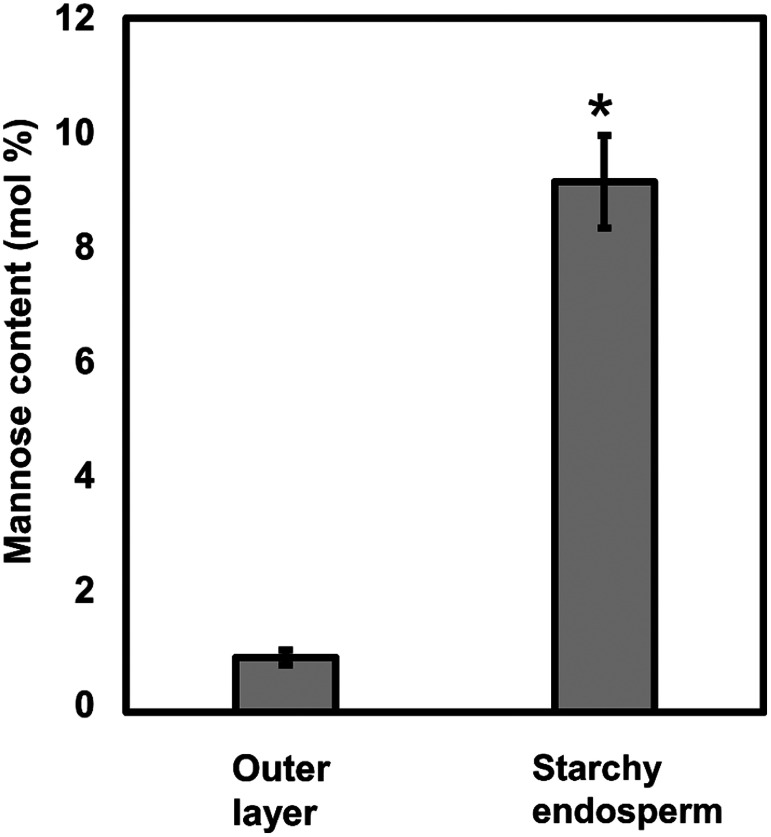
Figure 5. Mannose contents of the cell wall preparations obtained from the starchy endosperm and outer layer of glucomannan-containing rice grains. Cell walls were prepared from the starchy endosperm and outer layer, which is mainly composed of aleurone cells and lesser amount of seed coat and pericarp (Shibuya et al. 1985), of 4 glucomannan-containing varieties, Eiko, Kitaake, Matsumae and Michikogane. Bars indicate standard deviation. The asterisk indicates significant difference from the mannose contents of outer layer cell walls by Student’s *t*-test (*p*<0.01).

Aleurone cells are a part of endosperm and differentiated from the same fertilized polar nucleus which also becomes starchy endosperm during the grain maturation (Zheng and Wang 2014). As the results of differentiation, aleurone cell walls becomes much thicker than those of starchy endosperm cells (Figure 4B). Interestingly, the glucomannan in the aleurone cells of Fujisaka 5 was often detected in the middle portion of the cell walls (Figure 4B), suggesting the possibility that the glucomannan was synthesized in the early stage of endosperm development but the synthesis was downregulated during the secondary thickening process associated with the differentiation of aleurone layer. Detection of a much less amount of mannose as the component sugar of cell wall preparations from the outer layer of the mannose-containing rice grains, which is mainly composed of aleurone cells and lesser amount of seed coat and pericarp (Shibuya et al. 1985), compared to the starchy endosperm cell wall (Figure 5) coincides with this microscopic observation. Interestingly, Iglesias-Fernández et al. also immunologically detected the presence of mannan polysaccharides in the starchy endosperm cell walls, but not in the aleurone cell walls, of developing barley grains (Iglesias-Fernández et al. 2019). As these authors used light microscopy for their immunodetection of mannan polysaccharides, it might also be possible that they could not detect the presence of a small amount of mannan polysaccharides present in the middle portion of aleurone cell walls. These observations in rice and barley again suggest the possibility that the biosynthesis of cell wall (gluco)mannan in these grains occurs mainly at the early stage of endosperm development and is downregulated during the thickening of aleurone cell walls. On the other hand, Palmer et al. tried similar immunomicroscopic observation of developing rice grains with a β-linked mannan-specific antibody (LM21) but did not detect the presence of glucomannan (Palmer et al. 2015). The reason for this discrepancy with the present results is explained by the fact that the rice variety used in their study, “Koshihikari”, does not contain glucomannan in the starchy endosperm cell wall (Supplementary Figure S1A).

### Comparison of the starchy endosperm and callus cell walls suggested the tissue-specific accumulation of glucomannan in developing rice endosperm

**Figure 6:**
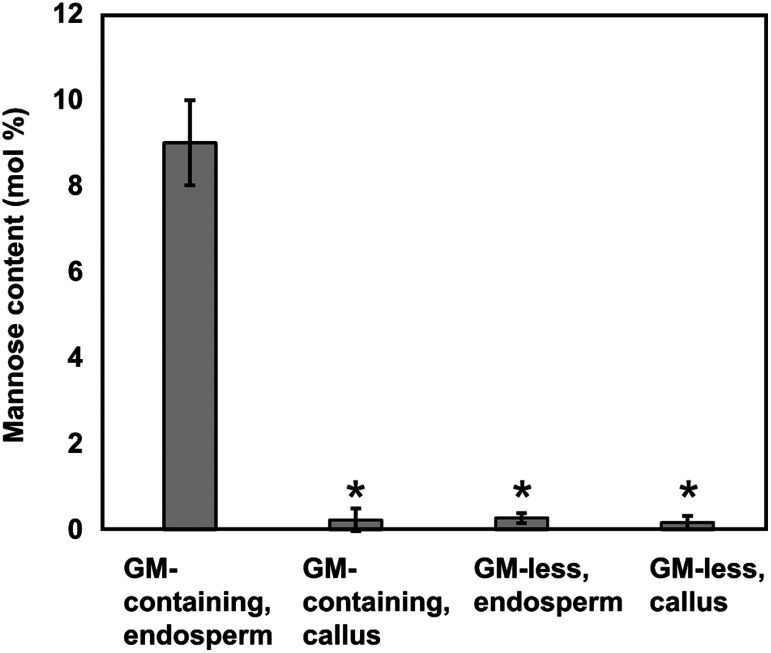
Figure 6. Mannose contents of the cell wall preparations obtained from the starchy endosperm and callus tissues from glucomannan-containing and glucomannan-less rice varieties. Cell walls were prepared from the starchy endosperm and callus cells of following rice varieties. Glucomannan(GM)-containing varieties; Fujisaka 5, Ishikari, Kitaake, Michikogane. Glucomannan(GM)-less varieties; Nipponbare, Reishikou, Yukihikari. Bars indicate standard deviation. The asterisks indicate significant differences from the mannose contents of glucomannan-containing endosperm cell walls by Student’s *t*-test (*p*<0.01).

As the accumulation of glucomannan was found in the thin starchy endosperm cell walls and often in the middle portion of the aleurone cell walls, we doubted whether the glucomannan accumulation could be a general event associated with thin, primary cell walls. To clarify this, we compared the amount of glucomannan between the starchy endosperm cell walls and callus cell walls of glucomannan-containing varieties. As shown in Figure 6, mannose contents of callus cell walls of 4 glucomannan-containing varieties were almost negligible, similar to the cell walls of starchy endosperm and callus of glucomannan-less varieties, indicating the accumulation of glucomannan is not an event generally associated with thin, primary cell walls but could be a tissue specific event.

### Comparative genomic analysis indicated the presence of striking differences between *OsCslA12* genes of glucomannan-positive and negative rice varieties, Kitaake and Nipponbare

**Figure 7:**
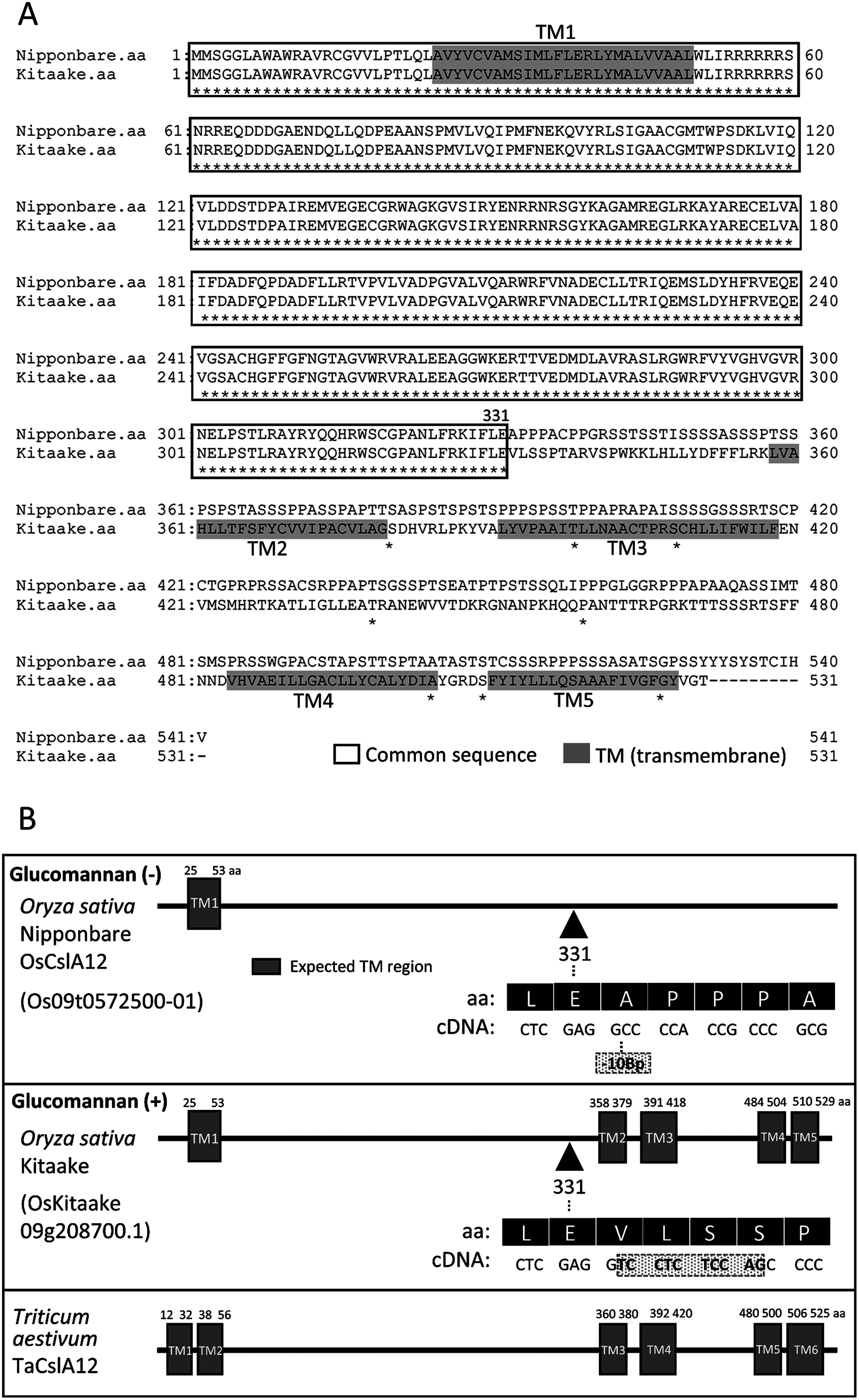
Figure 7. Comparison of OsCslA12 proteins between Nipponbare and Kitaake. A. Amino acid sequences of OsCslA12 of Nipponbare (Os09t0572500-01) and Kitaake (OsKitaake09g208700.1). Note that the amino acid sequences of these proteins are completely the same until 331 aa but the sequences after that are totally different, because of the frame shift caused by the deletion of 10 bases in the Nipponbare gene. Shadowed sequences indicate putative transmembrane domains (TMs) predicted by phobius (https://phobius.sbc.su.se/ (Accessed May 22, 2023)). Box indicates common sequences between Nippobare and Kitaake. Asterisks indicate the conserved residues in both sequences. B. Prediction of transmembrane domains in OsCslA12 proteins of Nippobare (Glucomannan (−)), Kitaake (Glucomannan (+)) and TaCslA12 of wheat, *Triticum aestivum*. Gray box indicates putative transmembrane domains (TMs). The amino acid (aa) and cDNA sequences near the region where the 10 bp deletion caused the frame shift in the Nipponbare OsCslA12 sequence are also shown.

Concerning to the biosynthesis of (gluco)mannan in higher plants, it has been known that cellulose synthase-like A (CslA) enzymes of Arabidopsis and other land plants catalyze the synthesis of (gluco)mannan (Voiniciuc 2022; Zhong et al. 2019). Previous genetic study also indicated that the gene regulating this glucomannan cell wall trait in rice resides on rice chromosome 9 but the gene itself was not identified (Yano et al. 1993, https://shigen.nig.ac.jp/rice/oryzabase/asset/rgn/vol10/vXIV27.htm). Survey of cellulose/mannan synthase-related gene(s) on rice chromosome 9 that showed noticeable differences between Kitaake and Nipponbare, which are the reference genome varieties and represent glucomannan positive and negative japonica varieties, respectively, indicated the presence of striking differences between the nucleotide sequences of *OsCslA12* gene, an ortholog of *TaCslA12* of wheat (Verhertbruggen et al. 2021) (Figure 7B and Supplementary Figure S4). While the amino acid sequence of OsCslA12 protein of Kitaake deduced from the nucleotide sequence showed a high similarity with that of TaCslA12, of which mannan synthase activity was confirmed in heterologous expression systems (Verhertbruggen et al. 2021), the amino acid sequence of Nipponbare OsCslA12 was quite different from that of Kitaake OsCslA12, especially in the C-terminal half region, because of the frame shift caused by the deletion of 10-base pairs in the coding region of *OsCslA12* gene (Figure 7A and Supplementary Table S1). The effect of such difference on the structure of OsCslA12 protein is also evident in the domain search of two proteins, where Kitaake OsCslA12 was predicted to contain 5 transmembrane domains, OsCslA12 of Nipponbare lacked most transmembrane domains except TM1 (Figure 7B). As the transmembrane domains lost in the Nipponbare OsCslA12 are conserved in TaCslA12 of wheat and indicated to be involved in the localization to Golgi membrane (Verhertbruggen et al. 2021), the lack of these transmembrane domains, as well as the completely different amino acid sequence in a part of the C-terminal half, indicates that the Nipponbare OsCslA12 protein is unable to localize and catalyze glucomannan synthesis at the Golgi apparatus. These observations suggested a possible scenario of the genetic basis of all-or-none trait of glucomannan cell wall in rice, i.e., the mutation at the *OsCslA12* gene caused the glucomannan-negative trait in these varieties.

## Discussion

Present results showed that, in addition to the previously reported cell wall polysaccharides, β-1,4-linked glucomannan is accumulated in the starchy endosperm and aleurone cell walls of rice grains in a variety-dependent manner. Comprehensive analysis of a wide range of rice varieties indicated the all-or-none distribution of the glucomannan cell wall trait among the rice varieties, suggesting that it is a qualitative trait controlled by the allelic differences of a single (or two) gene(s) (Kumar et al. 2017; St Clair 2010). This is very different from the genetic variation of arabinoxylan of cereal grains, of which contents showed a continuous distribution among wheat and barley varieties and have been recognized as a qualitative trait controlled by many more genes (Hassan et al. 2017; Hernández-Espinosa et al. 2020; Lovegrove et al. 2020; Marcotuli et al. 2015). In the glucomannan-containing varieties, glucomannan often accounts for 10% or more of the starchy endosperm cell wall (estimated from the mannose contents of the cell walls reported here and the neutral/acidic sugar contents previously reported for rice endosperm cell walls (Shibuya et al. 1985)). This glucomannan accumulation seemed to occur at the early developmental stage of endosperm based on the immunoelectron microscopic observation (Figure 4), as similar to the observation in the developing barley endosperm (Iglesias-Fernández et al. 2019). It was also suggested that the accumulation of glucomannan in the endosperm cell walls could be a tissue specific event, based on the comparison of the mannose contents between the endosperm cell walls and callus cell walls (Figure 6), though the point should further be confirmed. Concerning to the distribution of (gluco)mannans in other cereal grains, wheat and barley were reported to contain 2–3% of mannan/glucomannan in the endosperm cell walls (Burton and Fincher 2014).

It should be noted, on the other hand, Kosik et al. did not find such drastic difference in the mannose contents of endosperm cell wall preparations (“water-unextractable non-starchy polysaccharides” from polished rice) from 370 rice lines, including some varieties overlapping with the present study (Kosik et al. 2020). Most possible reason for the discrepancy between the results of these two studies seems to be the difference in the hydrolysis conditions used in these studies. While the two-step sulfuric acid hydrolysis developed for the hydrolysis of crystalline cellulose (Saeman et al. 1945) was used in the present study, Kosik et al. hydrolyzed the cell wall preparations with 2 M TFA, which is known to have limitations for the hydrolysis of cellulose, xyloglucan backbone (Talmadge et al. 1973), and may also for the hydrolysis of glucomannan because of the structural similarity of these β-1,4-linked polysaccharides. In fact, Kosik et al. detected galacturonic acid and galactose as the major components of their cell wall preparations, suggesting their results mainly reflect the sugar composition of pectic polysaccharides of rice endosperm cell wall.

The result of structural analysis of the purified rice glucomannan indicated a characteristic structural feature of the glucomannan. Different from the (galacto)glucomannans from several dicots and a gymnosperm (Fernandez and Murray 1986; Maeda et al. 1980; Yu et al. 2018, 2022), the rice glucomannan showed a much higher mannose/glucose ratio, more than 10, and also contained very small amount of galactose. The rice glucomannan contained a linear, consecutive β-1,4-linked mannan backbone and generated a series of β-1,4-linked mannooligosaccharides by mild acid hydrolysis (Figure 3A). This is a contrasting structural feature compared to the galactoglucomannans from the eudicot primary cell walls and Arabidopsis seed mucilage, which contained alternating repeats of Glc-Man in the backbone (Yu et al. 2018, 2022). Such structural feature of the rice glucomannan seems to relate to the observed strong binding to cellulose microfibril. Presence of the linear β-1,4-linked mannan backbone in the rice glucomannan coincides with the detection of glucomannan in the developing rice endosperm by the antibody against β-1,4-linked mannooligosaccharides (Figure 4). On the other hand, it should be pointed that some structural variations could be present between the glucomannans in the 4 M NaOH soluble fraction and α-cellulose fraction. Although we could not purify the glucomannan from the 4 M NaOH soluble fraction because of the presence of other hemicellulosic polysaccharides, higher Gal/Man ratio observed for this fraction (ca. 8 times higher than that of α-cellulose fraction, Table 1) seems to indicate the presence of glucomannan with more Gal side chains and weaker interaction with cellulose microfibril, though the point should be carefully evaluated considering the possible contribution of a small amount of galactose-containing pectic polysaccharides remaining in this fraction.

What could be the effect of glucomannan accumulation on the biological function of the cell wall? The fact that a significant amount of glucomannan remained in the α-cellulose fraction of starchy endosperm cell wall of rice, even after 4 M NaOH extraction, suggested that at least a part of the glucomannan is firmly associated with cellulose microfibril in the cell wall. It has been reported that the conformation of glucomannan is similar to that of cellulose and the glucomannan chains are strongly adsorbed at the micro/macrofibrillar surface in the cell walls (Moreira and Filho 2008; Peng et al. 2019; Shah et al. 2019). Physicochemical studies with bacterial cellulose and purified galactoglucomannan further indicated that the flexible and hydrated mannan segments can create aggregated layers that can bridge adjacent cellulose bundles, acting as a gluing agent between cellulose fibrils and bundles (Berglund et al. 2020). Thus, the presence of glucomannan in the cell wall could affect the supramolecular structure and physicochemical/biochemical properties of the starchy endosperm and aleurone cell walls of rice grain.

Accumulation of glucomannan during early development of rice endosperm could affect the maturation/germination process of rice grains through the changes in the properties of the cell wall. During the maturation of cereal grains such as rice and wheat, expansion of the starchy endosperm cells precedes the filling with starch and proteins (Calderini et al. 2021; del Rosario et al. 1968; Hoshikawa 1968). The size of the expanding endosperm cells, which is governed by turgor pressure and the extensibility of the cell wall, is a major factor determining the size of mature grains. For example, under high night temperature condition, expansion of starchy endosperm cells of rice decreases during the ripening stage and resulted in the decrease of the grain size (Morita et al. 2005; Wada et al. 2021). While the observed reduction of starchy endosperm cell expansion was mostly attributed to the osmotic adjustment, possible change of the cell wall extensibility was also suggested (Wada et al. 2021). In wheat, based on the assumption that the expansion of the cells in developing grains could increase the holding capacity for storage reserves and eventually crop yield, Calderini et al. recently generated a transgenic wheat that overexpressed an α-expansin under the control of wheat *PinB* promotor, which drives gene expression in starchy endosperm, aleurone and pericarp (Calderini et al. 2021). They observed the increase of grain size as well as grain yield for this transgenic wheat, avoiding a negative effect on grain number. In light of these observations, it seems possible that the presence/absence of glucomannan in starchy endosperm cell walls of rice could affect the growth of rice grains through the change of the physicochemical properties of the cell wall. On the other hand, it should also be noted that glucomannan and xyloglucan seem to play some redundant roles in the construction of primary cell walls and regulation of cell elongation (Yu et al. 2022). As rice endosperm cell wall contains significant amounts of xyloglucan (Shibuya and Misaki 1978), the effect of the lack of glucomannan on the cell wall properties and cell expansion might be partly compensated by the presence of xyloglucan.

Starchy endosperm of cereal grains is a major reserve of nutrients required for germination and seedling growth (Yan et al. 2014). During germination, these reserve materials are mobilized by corresponding hydrolases secreted from scutellum and aleurone cells and provided to the growing seedlings (Fincher 1989). Starchy endosperm cell walls are a potential barrier for the transport of these hydrolases to the site of mobilization of reserve materials. Supporting the notion, degradation of the starchy endosperm cell walls was observed during early germination process of barley and wheat (Fincher 1989; Gibbons 1980; Selvig et al. 1986). It has been recognized that cell wall degrading enzymes play roles for the loosening/penetrability changes of seed cell walls in the germination process (Fincher 1989; Rodriguez-Gacio Mdel et al. 2012; Steinbrecher and Leubner-Metzger 2017). Andriotis et al. observed a specific inhibitor of arabinoxylan arabinofuranohydrolase, 1,4-dideoxy-1,4-imino-L-arabinitol, reduced the degradation of arabinoxylan in the endosperm cell walls and starch degradation during germination of barley. Based on these results and other histological observations, the authors proposed that starch degradation in the endosperm is dependent on the cell wall degradation, which permeabilizes rapid diffusion of amylolytic enzymes (Andriotis et al. 2016). Accumulation of glucomannan in the starchy endosperm cell wall of rice could affect such germination process through the changes of the properties of the cell wall and the requirement of additional enzymes for the degradation of the cell wall. Interestingly, it was reported that β-mannanase activity as well as the expression of β-mannanase genes were up-regulated during the germination of rice (Ren et al. 2008; Yuan et al. 2007). Whether such observation relates to the degradation of the glucomannan during germination of the corresponding rice varieties remains for future studies.

From the viewpoint of rice grains as food and industrial materials, it is noteworthy that the presence/absence of glucomannan in the cell walls showed some characteristic distribution among the groups of rice varieties. For example, japonica varieties bred for the brewing of Japanese “Sake” often contained glucomannan in the starchy endosperm cell walls (Supplementary Figure S1E). Concerning to the relationships between Sake brewing and rice cell walls, it has been known that the degradation of starchy endosperm cell wall of steamed rice by the enzymes secreted by *Aspergillus oryzae* contributes to the efficient utilization of starch in “Sake” mash brewing by helping the penetration of fungal hyphae and enhancing the accessibility of starch degrading enzymes (Yamane et al. 2002a, 2002b). Cell wall rigidity may also affect the broken tolerance of rice grains, which could be a factor to obtain the core region of starchy endosperm for Sake fermentation efficiently. Also, many modern japonica varieties bred for better eating qualify for Japanese, i.e., giving rather soft and sticky cooked rice, tend not to contain glucomannan in the starchy endosperm cell walls (Supplementary Figure S1A, Figure 1C). It is interesting to see whether the presence/absence of glucomannan in the starchy endosperm cell wall affects the properties of cooked rice, as it was previously shown that the enzymatic modification of starchy endosperm cell wall significantly changed the cooking properties and texture of rice grains (Shibuya and Iwasaki 1984). On the other hand, speculations on the possible effects of glucomannan cell wall trait in the use of rice grains should be carefully evaluated because of the limited number of rice varieties discussed here and the presence of exceptions, e.g., presence of glucomannan-negative varieties among “Sake” brewing varieties. Whether the accumulation of glucomannan in the cell walls of rice varieties really relates to their applicability in specific usages should be validated by genetic approaches, such as the deletion/introduction of the gene responsible for this phenomenon in future studies.

Comparative genomic study between the two glucomannan-positive and negative varieties, Kitaake and Nipponbare, indicated that the mutation observed at the *OsCslA12* locus of Nipponbare, 10-base pair deletion in the cDNA, resulted in the generation of inactive glucomannan synthase and could be a reason of the glucomannan-negative phenotype. Interestingly, both of *CslA12* genes of wheat and rice have been reported to be specifically expressed in the endosperm (Verhertbruggen et al. 2021 for *TaCslA12*; RiceXpro Version 3.0 (https://ricexpro.dna.affrc.go.jp/ (Accessed May 22, 2023)) (Sato et al. 2013) for *OsCslA12* (Os09g0572500)). These results correspond well with the endosperm-specific accumulation of glucomannan in developing rice grains reported in this paper. On the other hand, it is not clear whether such differences in the *OsCslA12* locus are common to other glucomannan-positive and negative varieties. Whether any other genes required for the biosynthesis of glucomannan, such as the enzymes for the supply of GDP sugars and so on (Zhong et al. 2019), contribute to the glucomannan cell wall trait is not understood too. Finally, whether the mutation at the *OsCslA12* locus explains the all-or-none distribution of glucomannan in the rice endosperm cell walls should be evaluated by genetic studies such as the knockout of the wild type gene as well as the complementation of the mutant gene with the wild type gene. Finding the gene responsible for the glucomannan cell wall trait should lead the way to evaluate the contribution of such differences in the rice endosperm cell walls to the agronomic traits discussed in the present paper.

## Abbreviations

BSA: bovine serum albumin
Cello_4_-Sepharose: cellotetraosyl-Sepharose 6B
HPAEC: high-performance anion-exchange chromatography
Man_4_-antibody: mannooligosaccharide-specific antibody
Man_4_-BSA: mannotetraosyl-BSA
Man_(oligo)_-Sepharose: mannooligosyl-Sepharose 6B
OVA: ovalbumin
TFA: trifluoroacetic acid

## Description of Supplementary Files

Figure S1. Distribution of the mannose-positive cell wall in specific rice groups.

Figure S2. Fractionation of the partial acid hydrolysate of rice glucomannan on a Bio Gel P-2/P-4 column.

Figure S3. Binding specificity of the anti-mannooligosaccharide antibody.

Figure S4. Nucleotide sequences of *OsCslA12* cDNA of Nipponbare and Kitaake.

Table S1. Identity (similarity) in the amino acid sequences of CslA12s of rice (var. Nipponbare and Kitaake) and wheat.

## References

[RAndriotis2016] Andriotis VM, Rejzek M, Barclay E, Rugen MD, Field RA, Smith AM (2016) Cell wall degradation is required for normal starch mobilisation in barley endosperm. *Sci Rep* 6: 33215 27622597 10.1038/srep33215 PMC5020691

[RArsovski2010] Arsovski AA, Haughn GW, Western TL (2010) Seed coat mucilage cells of *Arabidopsis thaliana* as a model for plant cell wall research. *Plant Signal Behav* 5: 796–801 20505351 10.4161/psb.5.7.11773 PMC3014532

[RBaba1994] Baba K, Sone Y, Kaku H, Misaki A, Shibuya N, Itoh T (1994) Localization of hemicelluloses in the cell walls of some woody plants using immuno-gold electron microscopy. *Holzforschung* 48: 297–300

[RBassett2021] Bassett A, Hooper S, Cichy K (2021) Genetic variability of cooking time in dry beans (Phaseolus vulgaris L.) related to seed coat thickness and the cotyledon cell wall. *Food Res Int* 141: 109886 33641942 10.1016/j.foodres.2020.109886

[RBerglund2020] Berglund J, Mikkelsen D, Flanagan BM, Dhital S, Gaunitz S, Henriksson G, Lindstrom ME, Yakubov GE, Gidley MJ, Vilaplana F (2020) Wood hemicelluloses exert distinct biomechanical contributions to cellulose fibrillar networks. *Nat Commun* 11: 4692 32943624 10.1038/s41467-020-18390-z PMC7499266

[RBulone2019] Bulone V, Schwerdt JG, Fincher GB (2019) Co-evolution of enzymes involved in plant cell wall metabolism in the grasses. *Front Plant Sci* 10: 1009 31447874 10.3389/fpls.2019.01009 PMC6696892

[RBurton2014] Burton RA, Fincher GB (2014) Evolution and development of cell walls in cereal grains. *Front Plant Sci* 5: 456 25309555 10.3389/fpls.2014.00456 PMC4161051

[RCalderini2021] Calderini DF, Castillo FM, Arenas MA, Molero G, Reynold MP, Craze M, Bowden S, Milner MJ, Wallington EJ, Dowle A, et al. (2021) Overcoming the trade-off between grain weight and number in wheat by the ectopic expression of expansin in developing seeds leads to increased yield potential. *New Phytol* 230: 629–640 33124693 10.1111/nph.17048 PMC8048851

[RCapuano2019] Capuano E, Pellegrini N (2019) An integrated look at the effect of structure on nutrient bioavailability in plant foods. *J Sci Food Agric* 99: 493–498 30066376 10.1002/jsfa.9298

[RCarpita1993] Carpita NC, Gibeaut DM (1993) Structural models of primary cell walls in flowering plants: consistency of molecular structure with the physical properties of the walls during growth. *Plant J* 3: 1–30 8401598 10.1111/j.1365-313x.1993.tb00007.x

[RCosgrove2014] Cosgrove DJ (2014) Re-constructing our models of cellulose and primary cell wall assembly. *Curr Opin Plant Biol* 22: 122–131 25460077 10.1016/j.pbi.2014.11.001 PMC4293254

[Rdel1968] del Rosario AR, Briones VP, Vidal AJ, Juliano BO (1968) Composition and endosperm structure of developing and mature rice kernel. *Cereal Chem* 45: 225–235

[RFernandez1986] Fernandez EC, Murray LL (1986) Douglas-fir bark. II. Isolation and characterization of a glucomannan. *Wood Fiber Sci* 18: 436–445

[RFincher1989] Fincher GB (1989) Molecular and cellular biology associated with endosperm mobilization in germinating cereal grains. *Annu Rev Plant Physiol Plant Mol Biol* 40: 305–346

[RGalvez2011] Galvez-Lopez D, Laurens F, Quemener B, Lahaye M (2011) Variability of cell wall polysaccharides composition and hemicellulose enzymatic profile in an apple progeny. *Int J Biol Macromol* 49: 1104–1109 21939685 10.1016/j.ijbiomac.2011.09.007

[RGarcia2019] Garcia-Gimenez G, Russell J, Aubert MK, Fincher GB, Burton RA, Waugh R, Tucker MR, Houston K (2019) Barley grain (1,3;1,4)-beta-glucan content: effects of transcript and sequence variation in genes encoding the corresponding synthase and endohydrolase enzymes. *Sci Rep* 9: 17250 31754200 10.1038/s41598-019-53798-8 PMC6872655

[RGibbons1980] Gibbons GC (1980) On the sequential determination of α-amylase transport and cell wall breakdown in germinating seeds of Hordeum vulgare. *Carlsberg Res Commun* 45: 177–184

[RGrundy2016] Grundy MM, Edwards CH, Mackie AR, Gidley MJ, Butterworth PJ, Ellis PR (2016) Re-evaluation of the mechanisms of dietary fibre and implications for macronutrient bioaccessibility, digestion and postprandial metabolism. *Br J Nutr* 116: 816–833 27385119 10.1017/S0007114516002610 PMC4983777

[RHakomori1964] Hakomori S (1964) A rapid permethylation of glycolipid, and polysaccharide catalyzed by methylsulfinyl carbanion in dimethyl sulfoxide. *J Biochem* 55: 205–208 14135466

[RHassan2017] Hassan AS, Houston K, Lahnstein J, Shirley N, Schwerdt JG, Gidley MJ, Waugh R, Little A, Burton RA (2017) A Genome Wide Association Study of arabinoxylan content in 2-row spring barley grain. *PLoS One* 12: e0182537 28771585 10.1371/journal.pone.0182537 PMC5542645

[d66e1546] Hernández-Espinosa N, Romano GP, Crespo-Herrera L, Singh R, Guzmán C, Ibba MI (2020) Endogenous arabinoxylans variability in refined wheat flour and its relationship with quality traits. *J Cereal Sci* 95: 103062

[RHoshikawa1968] Hoshikawa K (1968) Studies on the development of endosperm in rice. 1. Process of endosperm tissue formation. *Jpn J Crop Sci* 36: 151–161

[RHouston2015] Houston K, Burton RA, Sznajder B, Rafalski AJ, Dhugga KS, Mather DE, Taylor J, Steffenson BJ, Waugh R, Fincher GB (2015) A genome-wide association study for culm cellulose content in barley reveals candidate genes co-expressed with members of the CELLULOSE SYNTHASE A gene family. *PLoS One* 10: e0130890 26154104 10.1371/journal.pone.0130890 PMC4496100

[RIglesias2019] Iglesias-Fernández R, Pastor-Mora E, Vicente-Carbajosa J, Carbonero P (2019) A possible role of the aleurone expressed gene HvMAN1 in the hydrolysis of the cell wall mannans of the starchy endosperm in germinating Hordeum vulgare L. seeds. *Front Plant Sci* 10: 1706 32038680 10.3389/fpls.2019.01706 PMC6983769

[RKall2007] Kall L, Krogh A, Sonnhammer EL (2007) Advantages of combined transmembrane topology and signal peptide prediction: The Phobius web server. *Nucleic Acids Res* 35(Web Server): W429–W432 17483518 10.1093/nar/gkm256 PMC1933244

[RKaur2017] Kaur S, Zhang X, Mohan A, Dong H, Vikram P, Singh S, Zhang Z, Gill KS, Dhugga KS, Singh J (2017) Genome-wide association study reveals novel genes associated with culm cellulose content in bread wheat (Triticum aestivum, L.). *Front Plant Sci* 8: 1913 29163625 10.3389/fpls.2017.01913 PMC5681534

[RKosik2020] Kosik O, Romero MV, Bandonill EH, Abilgos-Ramos RG, Sreenivasulu N, Shewry P, Lovegrove A (2020) Diversity of content and composition of cell wall-derived dietary fibre in polished rice. *J Cereal Sci* 96: 103122 10.3177/jnsv.65.S48 31619645

[RKumar2017] Kumar J, Gupta DS, Gupta S, Dubey S, Gupta P, Kumar S (2017) Quantitative trait loci from identification to exploitation for crop improvement. *Plant Cell Rep* 36: 1187–1213 28352970 10.1007/s00299-017-2127-y

[RKuno1999] Kuno A, Shimizu D, Kaneko S, Hasegawa T, Gama Y, Hayashi K, Kusakabe I, Taira K (1999) Significant enhancement in the binding of p-nitrophenyl-beta-D-xylobioside by the E128H mutant F/10 xylanase from Streptomyces olivaceoviridis E-86. *FEBS Lett* 450: 299–305 10359093 10.1016/s0014-5793(99)00498-6

[RKusakabe1977] Kusakabe I, Kawaguchl M, Yasui T, Kobayashl T (1977) Purification and some properties of extracellular xylanase from streptomyces sp. E-86. *(Nippon Nogeikagaku Kaishi)* 51: 429–437 (in Japanese)

[RLahaye2012] Lahaye M, Falourd X, Quemener B, Ralet MC, Howad W, Dirlewanger E, Arus P (2012) Cell wall polysaccharide chemistry of peach genotypes with contrasted textures and other fruit traits. *J Agric Food Chem* 60: 6594–6605 22697314 10.1021/jf301494j

[RLindberg1977] Lindberg B (1977) Recent advances in methods of isolating and purifying hemicelluloses. *Pure Appl Chem* 49: 1085–1093

[RLovegrove2020] Lovegrove A, Wingen LU, Plummer A, Wood A, Passmore D, Kosik O, Freeman J, Mitchell RAC, Hassall K, Ulker M, et al. (2020) Identification of a major QTL and associated molecular marker for high arabinoxylan fibre in white wheat flour. *PLoS One* 15: e0227826 32023285 10.1371/journal.pone.0227826 PMC7001892

[RMaeda1980] Maeda M, Shimahara H, Sugiyama N (1980) Detailed examination of the branched structure of konjac glucomannan. *Agric Biol Chem* 44: 245–252

[RManga2021] Manga-Robles A, Santiago R, Malvar RA, Moreno-Gonzalez V, Fornale S, Lopez I, Centeno ML, Acebes JL, Alvarez JM, Caparros-Ruiz D, et al. (2021) Elucidating compositional factors of maize cell walls contributing to stalk strength and lodging resistance. *Plant Sci* 307: 110882 33902850 10.1016/j.plantsci.2021.110882

[RMarcotuli2015] Marcotuli I, Houston K, Waugh R, Fincher GB, Burton RA, Blanco A, Gadaleta A (2015) Genome wide association mapping for Arabinoxylan content in a collection of tetraploid wheats. *PLoS One* 10: e0132787 26176552 10.1371/journal.pone.0132787 PMC4503733

[RMisaki1988] Misaki A, Kaku H, Sone Y, Shibata S (1988) Anti-α-L-arabinofuranose antibodies: Purification, immunochemical characterization and use in histochemical studies on plant cell wall polysaccharides. *Carbohydr Res* 173: 133–144

[RMoreira2008] Moreira LR, Filho EX (2008) An overview of mannan structure and mannan-degrading enzyme systems. *Appl Microbiol Biotechnol* 79: 165–178 18385995 10.1007/s00253-008-1423-4

[RMorita2005] Morita S, Yonemaru J, Takanashi J (2005) Grain growth and endosperm cell size under high night temperatures in rice (*Oryza sativa* L.). *Ann Bot* 95: 695–701 15655104 10.1093/aob/mci071 PMC4246861

[RMouille2006] Mouille G, Witucka-Wall H, Bruyant MP, Loudet O, Pelletier S, Rihouey C, Lerouxel O, Lerouge P, Hofte H, Pauly M (2006) Quantitative trait loci analysis of primary cell wall composition in Arabidopsis. *Plant Physiol* 141: 1035–1044 16714406 10.1104/pp.106.079384 PMC1489910

[RNguyen2020] Nguyen DT, Gomez LD, Harper A, Halpin C, Waugh R, Simister R, Whitehead C, Oakey H, Nguyen HT, Nguyen TV, et al. (2020) Association mapping identifies quantitative trait loci (QTL) for digestibility in rice straw. *Biotechnol Biofuels* 13: 165 33062051 10.1186/s13068-020-01807-8 PMC7545568

[RNishitani2015] Nishitani K, Demura T (2015) Editorial: An emerging view of plant cell walls as an apoplastic intelligent system. *Plant Cell Physiol* 56: 177–179 25673766 10.1093/pcp/pcv001

[ROzawa2009] Ozawa K (2009) Establishment of a high efficiency Agrobacterium-mediated transformation system of rice (*Oryza sativa* L.). *Plant Sci* 176: 522–527 26493142 10.1016/j.plantsci.2009.01.013

[RPalmer2015] Palmer R, Cornuault V, Marcus SE, Knox JP, Shewry PR, Tosi P (2015) Comparative in situ analyses of cell wall matrix polysaccharide dynamics in developing rice and wheat grain. *Planta* 241: 669–685 25416597 10.1007/s00425-014-2201-4 PMC4328131

[RPeng2019] Peng H, Salmen L, Stevanic JS, Lu J (2019) Structural organization of the cell wall polymers in compression wood as revealed by FTIR microspectroscopy. *Planta* 250: 163–171 30953149 10.1007/s00425-019-03158-7

[RPopovsky2017] Popovsky-Sarid S, Borovsky Y, Faigenboim A, Parsons EP, Lohrey GT, Alkalai-Tuvia S, Fallik E, Jenks MA, Paran I (2017) Genetic and biochemical analysis reveals linked QTLs determining natural variation for fruit post-harvest water loss in pepper (Capsicum). *Theor Appl Genet* 130: 445–459 27844114 10.1007/s00122-016-2825-9

[RRen2008] Ren Y, Bewley JD, Wang X (2008) Protein and gene expression patterns of endo-β-mannanase following germination of rice. *Seed Sci Res* 18: 139–149

[RRodriguez2012] Rodríguez-Gacio M del C, Iglesias-Fernández R, Carbonero P, Matilla ÁJ (2012) Softening-up mannan-rich cell walls. *J Exp Bot* 63: 3976–3988 22553284 10.1093/jxb/ers096

[RSaeman1945] Saeman JF, Bubl JL, Harris EE (1945) Quantitative saccharification of wood and cellulose. *Ind Eng Chem Anal Ed* 17: 35–37

[RSakai2013] Sakai H, Lee SS, Tanaka T, Numa H, Kim J, Kawahara Y, Wakimoto H, Yang CC, Iwamoto M, Abe T, et al. (2013) Rice Annotation Project Database (RAP-DB): An integrative and interactive database for rice genomics. *Plant Cell Physiol* 54: e6 23299411 10.1093/pcp/pcs183 PMC3583025

[RSarkar2009] Sarkar P, Bosneaga E, Auer M (2009) Plant cell walls throughout evolution: Towards a molecular understanding of their design principles. *J Exp Bot* 60: 3615–3635 19687127 10.1093/jxb/erp245

[RSato2013] Sato Y, Takehisa H, Kamatsuki K, Minami H, Namiki N, Ikawa H, Ohyanagi H, Sugimoto K, Antonio BA, Nagamura Y (2013) RiceXPro version 3.0: Expanding the informatics resource for rice transcriptome. *Nucleic Acids Res* 41(D1): D1206–D1213 23180765 10.1093/nar/gks1125 PMC3531122

[RSelvig1986] Selvig A, Aarnes H, Lie S (1986) Cell wall degradation of barley during germination. *J Inst Brew* 92: 185–187

[RShah2019] Shah R, Huang S, Pingali SV, Sawada D, Pu Y, Rodriguez M Jr, Ragauskas AJ, Kim SH, Evans BR, Davison BH, et al. (2019) Hemicellulose-cellulose composites reveal differences in cellulose organization after dilute acid pretreatment. *Biomacromolecules* 20: 893–903 30554514 10.1021/acs.biomac.8b01511

[RShibuya1984a] Shibuya N (1984) Phenolic acids and their carbohydrate esters in rice endosperm cell walls. *Phytochemistry* 23: 2233–2237

[RShibuya1993] Shibuya N (1993) Varietal differences of chemical structure of rice endosperm cell wall and their genetic analyses. *J Brew Soc Japan * 88: 910–913 (in Japanese)

[RShibuya1978a] Shibuya N, Iwasaki T (1978) Polysaccharides and glycoproteins in the rice endosperm cell wall. *Agric Biol Chem* 42: 2259–2266

[RShibuya1984b] Shibuya N, Iwasaki T (1984) Effect of cell wall degrading enzymes on the cooking properties of milled rice and the texture of cooked rice. *(Nippon Shokuhin Kogyo Gakkaishi)* 31: 656–660

[RShibuya1985a] Shibuya N, Iwasaki T (1985) Structural features of rice bran hemicellulose. *Phytochemistry* 24: 285–289

[RShibuya1978b] Shibuya N, Misaki A (1978) Structure of hemicellulose isolated from rice endosperm cell wall: Mode of linkages and sequences in xyloglucan, beta-glucan and arabinoxylan. *Agric Biol Chem* 42: 2267–2274

[RShibuya1984c] Shibuya N, Nakane R (1984) Pectic polysaccharides of rice endosperm cell walls. *Phytochemistry* 23: 1425–1429

[RShibuya1985b] Shibuya N, Nakane R, Yasui A, Tanaka K, Iwasaki T (1985) Comparative study on cell wall preparations from rice bran, germ and endosperm. *Cereal Chem* 62: 252–258

[RSt2010] St Clair DA (2010) Quantitative disease resistance and quantitative resistance Loci in breeding. *Annu Rev Phytopathol* 48: 247–268 19400646 10.1146/annurev-phyto-080508-081904

[RSteinbrecher2017] Steinbrecher T, Leubner-Metzger G (2017) The biomechanics of seed germination. *J Exp Bot* 68: 765–783 27927995 10.1093/jxb/erw428

[RTalmadge1973] Talmadge KW, Keegstra K, Bauer WD, Albersheim P (1973) The structure of plant cell walls: I. The macromolecular components of the walls of suspension-cultured sycamore cells with a detailed analysis of the pectic polysaccharides. *Plant Physiol* 51: 158–173 16658279 10.1104/pp.51.1.158 PMC367375

[RTsukada2002] Tsukada K, Ishizaka M, Fujisawa Y, Iwasaki Y, Yamaguchi T, Minami E, Shibuya N (2002) Rice receptor for chitin oligosaccharide elicitor does not couple to heterotrimeric G-protein: Elicitor responses of suspension cultured rice cells from Daikoku dwarf (d1) mutants lacking a functional G-protein alpha-subunit. *Physiol Plant* 116: 373–382

[RVan2018] Van de Wouwer D, Boerjan W, Vanholme B (2018) Plant cell wall sugars: Sweeteners for a bio-based economy. *Physiol Plant* 164: 27–44 29430656 10.1111/ppl.12705

[RVerhertbruggen2021] Verhertbruggen Y, Bouder A, Vigouroux J, Alvarado C, Geairon A, Guillon F, Wilkinson MD, Stritt F, Pauly M, Lee MY, et al. (2021) The TaCslA12 gene expressed in the wheat grain endosperm synthesizes wheat-like mannan when expressed in yeast and Arabidopsis. *Plant Sci* 302: 110693 33288007 10.1016/j.plantsci.2020.110693

[RVoiniciuc2022] Voiniciuc C (2022) Modern mannan: A hemicellulose’s journey. *New Phytol* 234: 1175–1184 35285041 10.1111/nph.18091

[RVoiniciuc2016] Voiniciuc C, Zimmermann E, Schmidt MH, Gunl M, Fu L, North HM, Usadel B (2016) Extensive natural variation in Arabidopsis seed mucilage structure. *Front Plant Sci* 7: 803 27375657 10.3389/fpls.2016.00803 PMC4894908

[RWada2021] Wada H, Chang FY, Hatakeyama Y, Erra-Balsells R, Araki T, Nakano H, Nonami H (2021) Endosperm cell size reduction caused by osmotic adjustment during nighttime warming in rice. *Sci Rep* 11: 4447 33627723 10.1038/s41598-021-83870-1 PMC7904791

[RWang2020] Wang X, Shi Z, Zhang R, Sun X, Wang J, Wang S, Zhang Y, Zhao Y, Su A, Li C, et al. (2020) Stalk architecture, cell wall composition, and QTL underlying high stalk flexibility for improved lodging resistance in maize. *BMC Plant Biol* 20: 515 33176702 10.1186/s12870-020-02728-2 PMC7659129

[RWood2018] Wood JA, Tan HT, Collins HM, Yap K, Khor SF, Lim WL, Xing X, Bulone V, Burton RA, Fincher GB, et al. (2018) Genetic and environmental factors contribute to variation in cell wall composition in mature desi chickpea (Cicer arietinum L.) cotyledons. *Plant Cell Environ* 41: 2195–2208 29532951 10.1111/pce.13196

[RYamaguchi2000] Yamaguchi T, Yamada A, Hong N, Ogawa T, Ishii T, Shibuya N (2000) Differences in the recognition of glucan elicitor signals between rice and soybean: Beta-glucan fragments from the rice blast disease fungus Pyricularia oryzae that elicit phytoalexin biosynthesis in suspension-cultured rice cells. *Plant Cell* 12: 817–826 10810152 10.1105/tpc.12.5.817 PMC139929

[RYamane2002a] Yamane Y, Fujita J, Izuwa S, Fukuchi K, Shimizu R, Hiyoshi A, Fukuda H, Mikami S, Kizaki Y, Wakabayashi S (2002a) Properties of cellulose-degrading enzymes from *Aspergillus oryzae* and their contribution to material utilization and alcohol yield in sake mash fermentation. *J Biosci Bioeng* 93: 479–484 16233235 10.1016/s1389-1723(02)80095-0

[RYamane2002b] Yamane Y, Fujita J, Shimizu R, Hiyoshi A, Fukuda H, Kizaki Y, Wakabayashi S (2002b) Production of cellulose- and xylan-degrading enzymes by a koji mold, aspergillus oryzae, and their contribution to the maceration of rice endosperm cell wall. *J Biosci Bioeng* 93: 9–14 16233157

[RYan2014] Yan D, Duermeyer L, Leoveanu C, Nambara E (2014) The functions of the endosperm during seed germination. *Plant Cell Physiol* 55: 1521–1533 24964910 10.1093/pcp/pcu089

[RYano1993] Yano M, Zamorski R, Saito A, Shibuya N (1993) A dominant gene controlling accumulation of glucomannan in the cell wall of rice endosperm. *Rice Genet Newsl* 10: 107

[RYoshida1990] Yoshida S, Kusakabe I, Matsuo N, Shimizu K, Yasui T, Murakami K (1990) Structure of rice-straw arabinoglucuronoxylan and specificity of Streptomyces xylanase toward the xylan. *Agric Biol Chem* 54: 449–457 1368510

[RYu2018] Yu L, Lyczakowski JJ, Pereira CS, Kotake T, Yu X, Li A, Mogelsvang S, Skaf MS, Dupree P, Li A (2018) The patterned structure of galactoglucomannan suggests it may bind to cellulose in seed mucilage. *Plant Physiol* 178: 1011–1026 30185440 10.1104/pp.18.00709 PMC6236596

[RYu2022] Yu L, Yoshimi Y, Cresswell R, Wightman R, Lyczakowski JJ, Wilson LFL, Ishida K, Stott K, Yu X, Charalambous S, et al. (2022) Eudicot primary cell wall glucomannan is related in synthesis, structure, and function to xyloglucan. *Plant Cell* 34: 4600–4622 35929080 10.1093/plcell/koac238 PMC9614514

[RYuan2007] Yuan JS, Yang X, Lai J, Lin H, Cheng ZM, Nonogaki H, Chen F (2007) The endo-beta-mannanase gene families in Arabidopsis, rice, and poplar. *Funct Integr Genomics* 7: 1–16 16897088 10.1007/s10142-006-0034-3

[RZhang2021] Zhang B, Gao Y, Zhang L, Zhou Y (2021) The plant cell wall: Biosynthesis, construction, and functions. *J Integr Plant Biol* 63: 251–272 33325153 10.1111/jipb.13055

[RZheng2014] Zheng Y, Wang Z (2014) Differentiation mechanism and function of the cereal aleurone cells and hormone effects on them. *Plant Cell Rep* 33: 1779–1787 25007781 10.1007/s00299-014-1654-z

[RZhong2019] Zhong R, Cui D, Ye ZH (2019) Secondary cell wall biosynthesis. *New Phytol* 221: 1703–1723 30312479 10.1111/nph.15537

