# Supplementary material for: Variety-dependent accumulation of glucomannan in the starchy endosperm and aleurone cell walls of rice grains and its possible genetic basis
Source: Plant Biotechnol (Tokyo). 2023 Dec 25;40(4):321–36. doi: 10.5511/plantbiotechnology.23.0809a (PMC10905567; doi:10.5511/plantbiotechnology.23.0809a)
Supplement: Supplementary Data [file plantbiotechnology-40-4-23.0809a-s001.pdf]

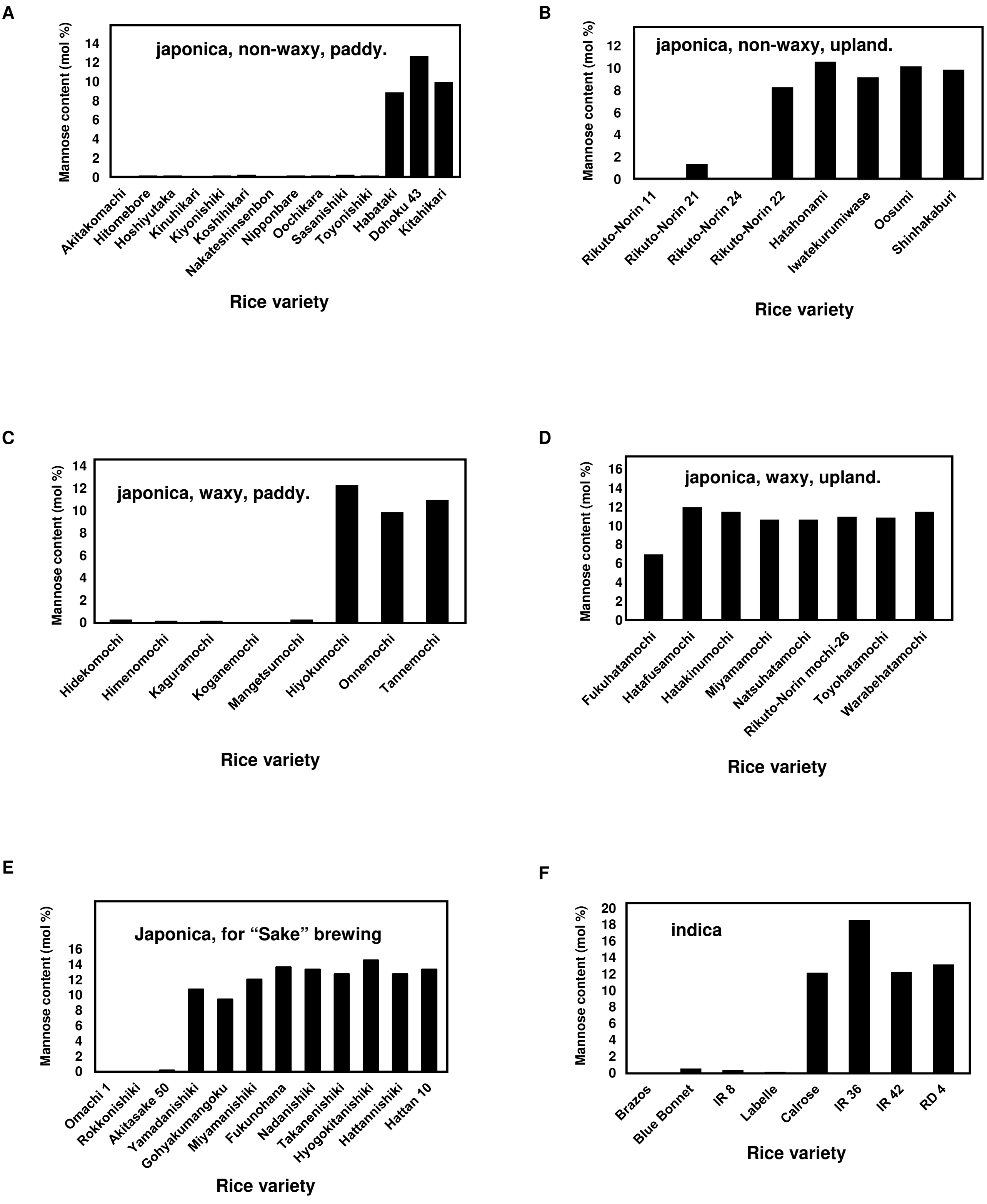

Supplementary Figure S1. Distribution of the mannose-positive cell wall in specific rice groups.

Endosperm cell wall preparations from arbitrary collected 58 rice varieties were analyzed for their neutral sugar composition after acid hydrolysis and their mannose contents are shown here. Rice varieties were categorized into 6 groups based on their subgroups (indica/japonica), waxy/non-waxy, upland/paddy and specific utilization (“Sake” brewing). Mannose content of each variety is a representative value.

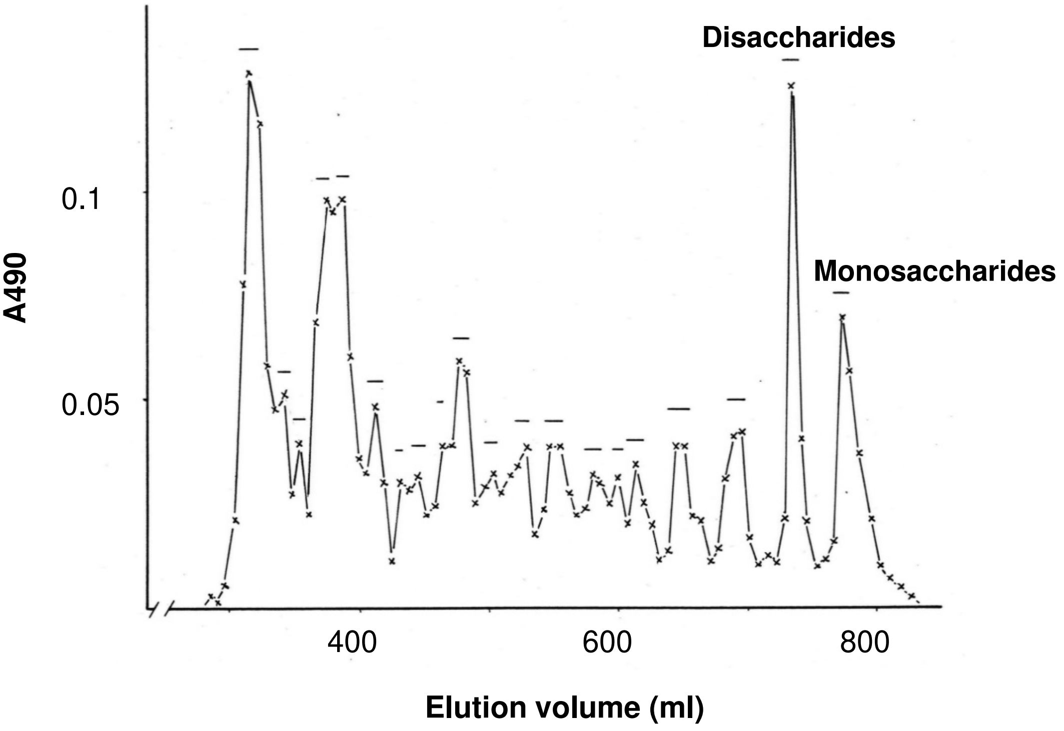

Supplementary Figure S2. Fractionation of the partial acid hydrolysate of rice glucomannan on a Biogel P-2/P-4 column.

Purified rice glucomannan was partially hydrolyzed by heating with 90% formic acid at 70°C for 30 min, followed by heating with 0.5 M TFA at 90°C for 1 h. Hydrolyzed samples were concentrated to dryness after each step and then redissolved with water. The partial acid hydrolyzates were subjected to gel filtration on a connected column of Bio Gel P-2 (2.6 x 88 cm) and P-4 (2.6 x 77 cm) and eluted with distilled water. Fractions were collected and analyzed for carbohydrate by phenol-sulfuric acid method. Disaccharide fraction obtained from the gel filtration and standard sugars were analyzed by HPAEC by using a gradient program suitable for oligosaccharide analyses.

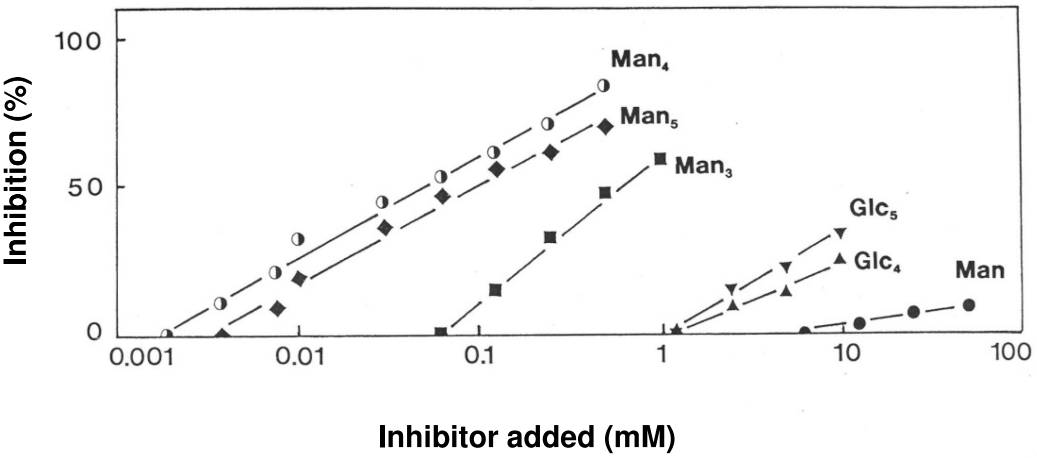

Supplementary Figure S3. Binding specificity of the anti-mannooligosaccharide antibody.

A 96-well flat bottom microtiter plate was coated with Man_4-BSA in 0.1 M carbonate buffer (pH 9.6). After blocking with 1% ovalbumin in the same buffer, 50 μl aliquots of the serially diluted Man_4-antibody (PBS/0.1% OVA/0.1% tween 20) were added to the wells and incubated at 37°C for 2 h. The antibody reacted with the Man_4-BSA was detected with the use of HRP-labeled goat anti-rabbit IgG. Hapten inhibition experiments were performed by the addition of the increasing amount of mono- and oligosaccharides to the microtiter wells.

```
Nipponbare    1:ATGATGAGCGGCGGGTTGGCATGGGCATGGCGGGCAGTGCGGTGCGGGGTGGTGTTGCCGACGCTGCAGCTGGCCGTCTA 80
Kitaake       1:ATGATGAGCGGCGGGTTGGCATGGGCATGGCGGGCAGTGCGGTGCGGGGTGGTGTTGCCGACGCTGCAGCTGGCCGTCTA 80
                ********************************************************************************

Nipponbare   81:CGTCTGCGTCGCCATGTCGATCATGCTCTTCCTCGAGCGACTCTACATGGCGCTCGTCGTCGCCGCCCTGTGGCTGATTC 160
Kitaake      81:CGTCTGCGTCGCCATGTCGATCATGCTCTTCCTCGAGCGACTCTACATGGCGCTCGTCGTCGCCGCCCTGTGGCTGATTC 160
                ********************************************************************************

Nipponbare  161:GCCGCCGCCGCCGCCGCAGCAACAGGAGGGAGCAGGACGACGATGGCGCTGAAAACGACCAGCTGCTGCAGGACCCGGAG 240
Kitaake     161:GCCGCCGCCGCCGCCGCAGCAACAGGAGGGAGCAGGACGACGATGGCGCTGAAAACGACCAGCTGCTGCAGGACCCGGAG 240
                ********************************************************************************

Nipponbare  241:GCGGCCAACAGCCCCATGGTCCTGGTGCAGATCCCCATGTTCAATGAGAAACAGGTTTACCGGCTTTCTATCGGTGCCGC 320
Kitaake     241:GCGGCCAACAGCCCCATGGTCCTGGTGCAGATCCCCATGTTCAATGAGAAACAGGTTTACCGGCTTTCTATCGGTGCCGC 320
                ********************************************************************************

Nipponbare  321:GTGTGGGATGACATGGCCATCTGACAAGCTGGTGATTCAGGTGCTGGATGACTCTACAGATCCAGCCATAAGGGAGATGG 400
Kitaake     321:GTGTGGGATGACATGGCCATCTGACAAGCTGGTGATTCAGGTGCTGGATGACTCTACAGATCCAGCCATAAGGGAGATGG 400
                ********************************************************************************

Nipponbare  401:TGGAGGGAGAGTGCGGGCGATGGGCGGGGAAGGGCGTGAGCATAAGGTACGAGAACAGGCGGAACAGGAGCGGGTACAAG 480
Kitaake     401:TGGAGGGAGAGTGCGGGCGATGGGCGGGGAAGGGCGTGAGCATAAGGTACGAGAACAGGCGGAACAGGAGCGGGTACAAG 480
                ********************************************************************************

Nipponbare  481:GCGGGGGCGATGCGGGAGGGGCTGCGGAAGGCGTACGCGAGGGAGTGTGAGTTGGTTGCCATCTTCGACGCAGACTTCCA 560
Kitaake     481:GCGGGGGCGATGCGGGAGGGGCTGCGGAAGGCGTACGCGAGGGAGTGTGAGTTGGTTGCCATCTTCGACGCAGACTTCCA 560
                ********************************************************************************

Nipponbare  561:GCCGGACGCCGACTTCCTCCTGCGCACAGTGCCGGTGCTGGTGGCGGACCCGGGGGTGGCGCTGGTGCAGGCGAGGTGGC 640
Kitaake     561:GCCGGACGCCGACTTCCTCCTGCGCACAGTGCCGGTGCTGGTGGCGGACCCGGGGGTGGCGCTGGTGCAGGCGAGGTGGC 640
                ********************************************************************************

Nipponbare  641:GGTTCGTGAACGCGGACGAGTGCCTTCTGACCCGCATCCAGGAGATGTCGCTGGACTACCACTTCCGCGTGGAGCAGGAG 720
Kitaake     641:GGTTCGTGAACGCGGACGAGTGCCTTCTGACCCGCATCCAGGAGATGTCGCTGGACTACCACTTCCGCGTGGAGCAGGAG 720
                ********************************************************************************

Nipponbare  721:GTGGGGTCGGCGTGCCACGGCTTCTTCGGGTTCAACGGCACGGCGGGCGTGTGGCGGGTGCGAGCCCTGGAGGAGGCGGG 800
Kitaake     721:GTGGGGTCGGCGTGCCACGGCTTCTTCGGGTTCAACGGCACGGCGGGCGTGTGGCGGGTGCGAGCCCTGGAGGAGGCGGG 800
                ********************************************************************************

Nipponbare  801:CGGGTGGAAGGAGCGGACGACGGTGGAGGACATGGACCTGGCGGTGCGAGCGAGCCTGAGGGGGTGGCGGTTCGTGTACG 880
Kitaake     801:CGGGTGGAAGGAGCGGACGACGGTGGAGGACATGGACCTGGCGGTGCGAGCGAGCCTGAGGGGGTGGCGGTTCGTGTACG 880
                ********************************************************************************

Nipponbare  881:TGGGGCACGTCGGGGTGCGGAACGAGCTGCCCAGCACGCTGCGCGCGTACCGGTACCAGCAGCACCGGTGGTCGTGCGGC 960
Kitaake     881:TGGGGCACGTCGGGGTGCGGAACGAGCTGCCCAGCACGCTGCGCGCGTACCGGTACCAGCAGCACCGGTGGTCGTGCGGC 960
                ********************************************************************************

Nipponbare  961:CCCGCCAACCTGTTCCGCAAGATTTTCCTCGAGG----------CCCCACCGCCCGCGTGTCCCCCTGGAAGAAGCTCCA 1030
Kitaake     961:CCCGCCAACCTGTTCCGCAAGATTTTCCTCGAGGTCCTCTCCAGCCCCACCGCCCGCGTGTCCCCCTGGAAGAAGCTCCA 1040
                **********************************          ************************************

Nipponbare 1031:CCTCCTCTACGATTTCTTCTTCCTCCGCAAGCTCGTCGCCCACCTCCTCACCTTCTCCTTCTACTGCGTCGTCATCCCCG 1110
Kitaake    1041:CCTCCTCTACGATTTCTTCTTCCTCCGCAAGCTCGTCGCCCACCTCCTCACCTTCTCCTTCTACTGCGTCGTCATCCCCG 1120
                ********************************************************************************

Nipponbare 1111:CCTGCGTCCTCGCCGGCTCCGACCACGTCCGCCTCCCCAAGTACGTCGCCCTCTACGTCCCCGCCGCCATCACCCTCCTC 1190
Kitaake    1121:CCTGCGTCCTCGCCGGCTCCGACCACGTCCGCCTCCCCAAGTACGTCGCCCTCTACGTCCCCGCCGCCATCACCCTCCTC 1200
                ********************************************************************************

Nipponbare 1191:AACGCCGCCTGCACCCCGCGCTCCTGCCATCTCCTCATCTTCTGGATCCTCTTCGAGAACGTCATGTCCATGCACCGGAC 1270
Kitaake    1201:AACGCCGCCTGCACCCCGCGCTCCTGCCATCTCCTCATCTTCTGGATCCTCTTCGAGAACGTCATGTCCATGCACCGGAC 1280
                ********************************************************************************

Nipponbare 1271:CAAGGCCACGCTCATCGGCCTGCTCGAGGCCACCCGCGCCAACGAGTGGGTCGTCACCGACAAGCGAGGCAACGCCAACC 1350
Kitaake    1281:CAAGGCCACGCTCATCGGCCTGCTCGAGGCCACCCGCGCCAACGAGTGGGTCGTCACCGACAAGCGAGGCAACGCCAACC 1360
                ********************************************************************************

Nipponbare 1351:CCAAGCACCAGCAGCCAGCTAATACCACCACCAGGCCTGGGAGGAAGACCACCACCAGCTCCAGCCGCACAAGCTTCTTC 1430
Kitaake    1361:CCAAGCACCAGCAGCCAGCTAATACCACCACCAGGCCTGGGAGGAAGACCACCACCAGCTCCAGCCGCACAAGCTTCTTC 1440
                ********************************************************************************

Nipponbare 1431:AATAATGACGTCCATGTCGCCGAGATCCTCCTGGGGGCCTGCCTGCTCTACTGCGCCCTCTACGACATCGCCTACGGCCG 1510
Kitaake    1441:AATAATGACGTCCATGTCGCCGAGATCCTCCTGGGGGCCTGCCTGCTCTACTGCGCCCTCTACGACATCGCCTACGGCCG 1520
                ********************************************************************************

Nipponbare 1511:CGACAGCTTCTACATCTACCTGCTCCTCCAGTCGGCCGCCGCCTTCATCGTCGGCTTCGGCTACGTCGGGACCTAGCAGC 1590
Kitaake    1521:CGACAGCTTCTACATCTACCTGCTCCTCCAGTCGGCCGCCGCCTTCATCGTCGGCTTCGGCTACGTCGGGACCTAG---- 1596
                ****************************************************************************

Nipponbare 1591:TACTACTACTCTTACTCTACATGCATCCATGTATAA                                             1626
Kitaake    1596:------------------------------------
```

Supplementary Figure S4. Nucleotide sequences of *OsCslA12* cDNA of Nipponbare and Kitaake.

Nucleotide sequences of Nipponbare *OsCslA12* (Os09t0572500-01) and Kitaake *OsCslA12* (OsKitaake09g208700.1) were obtained from The Rice Annotation Project Database (RAP-DB, https://rapdb.dna.affrc.go.jp/) and *Oryza* sativa Kitaake v3.1 database in Phytozome ver. 13 (https://phytozome-next.jgi.doe.gov/) respectively. Red letters indicate the position where the 10 base deletion was observed in Nipponbare.

Supplementary Table S1. Identity (similarity) in the amino acid sequences of CslA12s of rice (var. Nipponbare and Kitaake) and wheat.

	Whole protein	1-331aa	332aa-end
**Nipponbare OsCslA12** (Os09t0572500-01) **/** **Kitaake OsCslA12** (OsKitaake09g208700.1)	66.4% (73.4%)	100% (100%)	16.7% (55.6%)
**Nipponbare OsCslA12** (Os09t0572500-01) **/** **TaCslA12**	53.6% (68.4%)	78.7% (89.8%)	3.4% (9.5%)
**Kitaake OsCslA12** (OsKitaake09g208700.1) **/** **TaCslA12**	72.4% (87.8%)	78.7% (89.8%)	62.1% (83.8%)
